# Standardized Incidence Rate, Risk and Survival Outcomes of Second Primary Malignancy Among Renal Cell Carcinoma Survivors: A Nested Case-Control Study

**DOI:** 10.3389/fonc.2021.716741

**Published:** 2021-07-30

**Authors:** Zhixian Wang, Yisheng Yin, Jing Wang, Yunpeng Zhu, Xing Li, Xiaoyong Zeng

**Affiliations:** ^1^Department of Urology, Tongji Hospital, Tongji Medical College, Huazhong University of Science and Technology, Wuhan, China; ^2^Institute of Urology of Hubei Province, Wuhan, China

**Keywords:** kidney malignancy, SEER, second primary malignancy, surveillance, prognosis, standardized incidence rate

## Abstract

**Purpose:**

Second primary malignancy (SPM) is challenging for treatment and long-term survival. We sought to investigate the standardized incidence rate (SIR), risk factors, and survival outcomes for SPM after renal cell carcinoma (RCC) treatment.

**Method:**

A nested case-control study was designed, we identified all T1-4N0-1M0 RCC patients diagnosed between 2004 and 2015 in the Surveillance, Epidemiology, and End Results database and followed them for SPM diagnosis for up to 13 years. Patients with SPM diagnosis ≥6 months after treatment of primary T1-4N0-1M0 RCC were identified as the case cohort and SPM-free patients were the control cohort. SIRs and the excess risk were calculated. A competing risks and Cox model were used to evaluate the risk factors of SPM and overall survival (OS).

**Results:**

A cohort of 6,204 RCC patients with SPM were matched with a control group of 31,020 RCC patients without SPM. The median time-to-SPM interval was 54.5 months in RCC patients with SPM diagnosis. Besides, an SPM of T3/4 or/and M1 stage diagnosis was positively associated with a longer time-to-SPM interval. SIR of SPM increased by follow-up time and decreased with age at diagnosis (P_for all <_0.001). SPM in the kidney had the highest SIR (54.6, P <0.001) among all SPMs. Prostate cancer (29.8%) in males and breast cancer (23.5%) in females were the most common SPM. Older age, black ethnicity, male sex, higher family income, papillary RCC, and lower TNM stage were significant risk factors for SPM diagnosis. The proportion of deaths from SPM exceeds that of deaths from RCC 3 years after the first RCC treatment. Patients with SPM and early time-to-SPM interval shortens the OS compared with SPM-free patients. The 5-year OS was 85.9% and 58.9% from the first RCC and the SPM diagnosis, respectively. Besides, patients with low-grade/early-stage SPM could benefit from aggressive surgical treatment for solid tumors.

**Conclusions:**

Collectively, our study described the epidemiological characteristics of SPM among RCC survivors and identified the independent predictors of the SPM diagnosis and its survival outcomes. This study highlights the importance of patient education and follow-up after the surgery for RCC.

## Introduction

The burden of cancer worldwide is challenging. By 2040, the number of new cancer cases per year is expected to increase to 29.5 million and the number of cancer-related deaths to increase to 16.4 million (1). Renal cell carcinoma (RCC) is one of the top-10 most prevalent cancers and accounts for 3% and 5% of all malignancies in females and males, respectively (2). The latest data highlight that, in 2020, ~73,750 new cases of RCC and renal–pelvis cancer were diagnosed in the USA, and that ~14,830 patients died from RCC and renal–pelvis cancer, respectively (2).

Worldwide, there has been a 2% increase in RCC prevalence over the past two decades; this increase is particularly evident in developed countries (3). Currently, cancer can be detected at an earlier stage and an increase in overall survival (OS) experienced due to screening programs and improvements in technology, treatments, and supportive care (4–7). Even patients with advanced cancer can obtain increased OS as a result of treatment advances (8).

As of January 2019, there were an estimated 16.9 million cancer survivors in the USA, and the number of cancer survivors is projected to increase to 22.2 million by 2030 (1). One of the most life-threatening sequelae is the onset of a new cancer type for cancer survivors. There will be a substantial increase in the number of survivors being diagnosed with a second primary malignancy (SPM) due to longer survival and follow-up. It is not unusual for patients to experience multiple primary tumors during a lifetime (8). SPM has been documented in ~16% of cancers reported by the Surveillance, Epidemiology, and End Results (SEER) Program of the US National Cancer Institute (Bethesda, MD, USA) (7).

The risk of SPM could be increased by interactions between common etiological factors, particularly an unhealthy lifestyle (e.g., long-term tobacco use, excessive intake of alcohol), genetic susceptibility, environmental exposure, and patient factors (7, 9). In addition, research has shown that SPM can develop as a result of the late effects of certain treatments [e.g., radiotherapy, chemotherapy (10, 11)].

SPM brings challenges to the diagnosis and treatment of the disease. SPM must be differentiated from metastasis of primary cancer, and the treatment plan will be completely different. Metastatic tumors can be treated according to the latest clinical guidelines or clinical trials. If a primary malignancy is present with SPM, a strategy for anti-cancer treatment that can cover the two types of cancer without increasing the toxicity of the drug must be created and, simultaneously, the related pharmacologic effects must not affect the overall outcome negatively. However, most of the recruiting populations of clinical-trial research programs usually exclude patients with a history of cancer or SPM. Therefore, in daily clinical practice, it is important to recognize these issues and detection of SPM in time because this has a relevant impact on subsequent strategies for treatment management and OS prediction.

Here, we screened the SEER database to assess the incidence, risk, and survival outcomes of SPM among RCC survivors.

## Patients and Methods

### Data Source

All data were retrieved from the SEER database (https://seer.cancer.gov/) compiled by the US National Cancer Institute. The SEER database features 18 registries; collectively, these registries represent approximately 28% of the population of the USA. The characteristics of the patients identified were comparable to the general population. These data recorded the demographic and clinical characteristics of patients, cancer incidence, treatments, and survival outcomes from each cancer registry. The MP session of SEER *Stat software (version 8.3.6, https://seer.cancer.gov/seerstat/) was used to extract the detailed data of SPMs. In MP data, we were able to collate data relating to the sequence of multiple primary malignancies and the time duration associated with their occurrence. Since SEER databases were anonymized and were not associated with research studies. Consequently, the need for ethics approval was waived by the Ethics Review Board.

### Patient Identification

**Figure 1:**
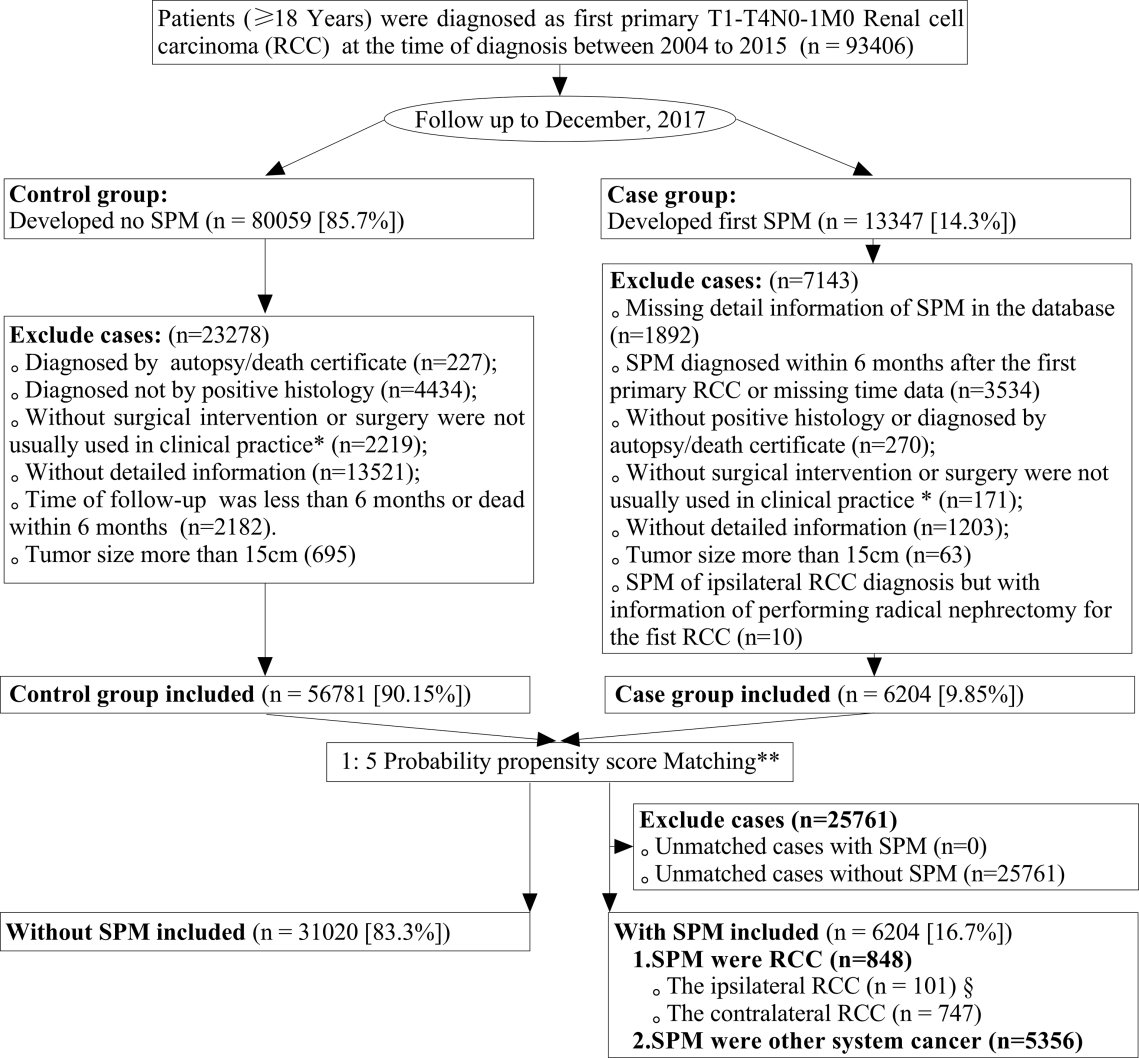
Flowchart showing the process used to screen data. SPM, second primary malignancy (SPM); RCC, renal cell carcinoma. *Patients undergoing cryosurgery/radiofrequency ablation (RX Summ–Surg Prim Site codes 13, 15, and 23, in the SEER database) and partial/radical nephrectomy (RX Summ–Surg Prim Site codes 30, 40, 50, 70, and 80, in the SEER database) were included in this study; **The year of the diagnosis of the first primary RCC and SEER registers were adjusted variables and used to merge the propensity score for patients by a logistic regression model with a case: control ratio of 1:5; §From 101 patients, 82 T1aN0M0 renal cell carcinomas were included for further analyses, and we excluded other 16 RCCs of tumor size >4 cm and three cases classified as T3a RCCs.

This was a nested case-control study. We prospectively identified patients who had been treated for primary T1-4N0-1M0 RCC from 2004 to 2015, and who then went on to develop SPM or SPM-free when the follow-up to December 2017. **Figure 1** is a flowchart that describes how patients and data were selected from the SEER database. Briefly, we included patients aged ≥18 years who had been treated for their first primary T1-4N0-1M0 RCC and who did not have any other organ metastatic diseases. All diagnoses of RCC and SPM had to have been confirmed by histology and not by autopsy or death certification. To avoid the inclusion of synchronous cancers in the case cohort, patients with pre-existing cancers at the diagnosis of RCC were excluded in our present analysis. In addition, only SPMs included in the MP data set that was pathologically proven and developed at least 6 months after the diagnosis of the first primary RCC were extracted. If the selected individuals had experienced multiple incidences of SPM, we retrieved detailed information relating only to the first occurrence of SPM. The site of the primary cancer was identified by reference to the *International Classification of Diseases for Oncology, 3rd Edition* (ICD-O-3).

### Study Variables

We collated a wide range of demographical and clinical variables, as follows: year of diagnosis, age at diagnosis, gender, race, or ethnicity (White and Others [Black, American Indian/Alaska Native, Asian Native, and Asian/Pacific Islander]), marital status, family income quartile, population, and region. We also collated a range of data related to tumors, as follows: tumor size (cm), histological cell type for RCC (clear cell, and non-clear cell [papillary and chromophobe]); tumor grade (well-differentiated [grade I], moderately differentiated [grade II], poorly differentiated [grade III], and undifferentiated [grade IV]); and American Joint Committee on Cancer (AJCC) (6^th^ edition, 2004-2009)/(7^th^ edition, 2010-2015) tumor node metastasis (TNM) staging classification. The surgical intervention included partial or radical nephrectomy, cryosurgery, or radiofrequency ablation.

### Statistical Analyses

Continuous variables are described as the mean ± standard deviation (SD) if they had a normal distribution and were compared using the Student’s *t*-test. Continuous variables that did not have a normal distribution are described as the median and interquartile range (IQR) and were compared using the Wilcoxon rank-sum test. Categorical variables are presented as frequencies (%) and were compared using the chi-squared test.

To control the bias related to selection between cases and control groups, the year and the first SEER registry of primary RCC were designated as adjusted covariables to ensure that the case and control cohorts were diagnosed during the same latency period, and to control for differences across different registries. In the present analysis, we undertook matching of the propensity score (1:5 for cases: controls) using the “nearest neighbor” method and the “MatchIt” package in R 3.6.3 for Windows (R Project for Statistical Computing, Vienna, Austria; www.r-project.org).

One of the primary objectives of this study was to investigate the standardized incidence rate (SIR), SIR is usually used to determine if the occurrence of a new disease in a relatively small population is high or low, and are being compared to disease rates in a reference population, usually the general population of the geographic area from which the cohort was selected. The adjusted SIR—along with its 95% confidence interval (CI)—was calculated as the ratio of SPM in patients diagnosed with primary RCC to the number of expected events in the general population (observed cases/expected cases) (12). Age, time interval, and tumor site were adjusted for the SIR calculation, and the significance of SIR was assessed using a likelihood ratio test. Person-years at risk for SPM development was computed for SPM site from the date of diagnosis of RCC to the date of diagnosis of an SPM, date of death, date of loss to follow-up, or end of the study period (13); furtherly, the excess risk per 10,000 person-year of SPM was calculated using the following formula: “[(observed cases - expected cases)*10,000/person-years at risk]”, SEER*Stat software was used for the analysis (13).

The risk factors and survival outcomes associated with SPM following a primary diagnosis of RCC were another concern. For the risk predictors of SPMs analysis, the SPM were stratified as kidney cancer (including contralateral and ipsilateral kidney cancer) and other non-kidney malignancies. We used a Fine and Gray competing-risks regression model to evaluate the risk factors for the first occurrence of SPM, and calculated the Sub-distribution hazard ratio (sHR) with 95%CIs for all risk factors. For Fine and Gray competing-risks regression analysis, we defined the “time interval” is the date from the diagnosis of primary RCC to the earliest date of the SPM diagnosis (event observed) or the final follow-up in December 2017 or dead (censored).

When investigating the risk factors associated with survival outcomes, we used Cox proportional hazards regression to analyze the risk factors for overall mortality from the first RCC diagnosis and first SPM diagnosis. Patients who died from RCC were identified as RCC-cause mortality, those who died from other causes were designated as ‘competing events’ before RCC-cause mortality. Any cause of death was considered as all-cause mortality; this was considered as a competing event for the occurrence of SPM. The time interval between RCC diagnosis and SPM, and the duration of survival, were defined as the time elapsed from the date of RCC diagnosis to the date of SPM diagnosis, and death or last contact, respectively.

All analyses were conducted using R 3.6.3. and P<0.05 (two-sided) was considered significant.

## Results

### Patients and Baseline Characteristics

A total of 93,406 patients were diagnosed with first primary T1-4N0-1M0 RCC from 2004 to 2015. During follow-up to December 2017, 13,347 of these patients (14.3%) reported SPM. Finally, we identified 6,204 patients (16.7%) in the case group and 31,020 patients (83.3%) in the control group after matching by the year of the diagnosis and different registries. **Supplementary Table 1** and **Table 1** list the baseline characteristics before and after propensity-score matching, respectively. In comparison with non-SPM patients, those with SPM: were older; more of them were male; were of black ethnicity; had greater papillary histology; were more likely to have tumor grade I/II, more likely to have been treated by cryosurgery/radiofrequency and partial neurectomy, and to have a smaller tumor size. SPM was more common in married patients, and less common in patients with a low family income. **Supplementary Table 2** lists the demographic and clinical characteristics for different case-control groups (non-RCC SPM *vs.* non-SPM; contralateral RCC SPM *vs.* non-SPM; ipsilateral RCC SPM *vs.* non-SPM).

**Table 1:** Demographic and clinic characteristics for patients with renal cell carcinoma as the first primary malignant tumor between 2004–2015 after propensity score matching*.

		Nested Case-Control		
	Overall	None-SPM (Control)	With SPM (Case)	*P-*value
	(N=37224)	(N=31020)	(N=6204)	
**Year at diagnosis**				1.000
2004	4068 (10.9%)	3382 (10.9%)	686 (11.1%)	
2005	4139 (11.1%)	3448 (11.1%)	691 (11.1%)	
2006	4432 (11.9%)	3708 (12.0%)	724 (11.7%)	
2007	4251 (11.4%)	3535 (11.4%)	716 (11.5%)	
2008	4216 (11.3%)	3513 (11.3%)	703 (11.3%)	
2009	3836 (10.3%)	3199 (10.3%)	637 (10.3%)	
2010	3150 (8.5%)	2625 (8.5%)	525 (8.5%)	
2011	2562 (6.9%)	2135 (6.9%)	427 (6.9%)	
2012	2202 (5.9%)	1835 (5.9%)	367 (5.9%)	
2013	2070 (5.6%)	1725 (5.6%)	345 (5.6%)	
2014	1470 (3.9%)	1225 (3.9%)	245 (3.9%)	
2015	828 (2.2%)	690 (2.2%)	138 (2.2%)	
**SEER registries**				0.979
Atlanta (Metropolitan)	1252 (3.4%)	1047 (3.4%)	205 (3.3%)	
California excluding SF/SJM/LA	7512 (20.2%)	6246 (20.1%)	1266 (20.4%)	
Connecticut	1752 (4.7%)	1473 (4.7%)	279 (4.5%)	
Detroit (Metropolitan)	2139 (5.7%)	1792 (5.8%)	347 (5.6%)	
Greater Georgia	2891 (7.8%)	2414 (7.8%)	477 (7.7%)	
Hawaii	566 (1.5%)	463 (1.5%)	103 (1.7%)	
Iowa	1880 (5.1%)	1554 (5.0%)	326 (5.3%)	
Kentucky	3102 (8.3%)	2594 (8.4%)	508 (8.2%)	
Los Angeles	3279 (8.8%)	2742 (8.8%)	537 (8.7%)	
Louisiana	2668 (7.2%)	2226 (7.2%)	442 (7.1%)	
New Jersey	4231 (11.4%)	3494 (11.3%)	737 (11.9%)	
New Mexico	462 (1.2%)	385 (1.2%)	77 (1.2%)	
Rural Georgia	72 (0.2%)	60 (0.2%)	12 (0.2%)	
San Francisco Oakland SMSA	1623 (4.4%)	1363 (4.4%)	260 (4.2%)	
San Jose Monterey	820 (2.2%)	676 (2.2%)	144 (2.3%)	
Seattle (Puget Sound)	2318 (6.2%)	1946 (6.3%)	372 (6.0%)	
Utah	657 (1.8%)	545 (1.8%)	112 (1.8%)	
**Marital status**				<0.001
Married	24730 (66.4%)	20459 (66.0%)	4271 (68.8%)	
Single/unmarried	5458 (14.7%)	4685 (15.1%)	773 (12.5%)	
Widowed/Divorced/Separated	7036 (18.9%)	5876 (18.9%)	1160 (18.7%)	
**Population density**				0.270
Counties	32546 (87.4%)	27103 (87.4%)	5443 (87.7%)	
Rural	603 (1.6%)	517 (1.7%)	86 (1.4%)	
Urban	4075 (10.9%)	3400 (11.0%)	675 (10.9%)	
**Region**				0.995
East	15968 (42.9%)	13308 (42.9%)	2660 (42.9%)	
Northern Plains	4019 (10.8%)	3346 (10.8%)	673 (10.8%)	
Pacific Coast	16118 (43.3%)	13436 (43.3%)	2682 (43.2%)	
Southwest	1119 (3.0%)	930 (3.0%)	189 (3.0%)	
**The median family income quartile**				0.016
1 or less	8158 (21.9%)	6882 (22.2%)	1276 (20.6%)	
1 ~ 2	9753 (26.2%)	8135 (26.2%)	1618 (26.1%)	
2 ~ 3	9782 (26.3%)	8079 (26.0%)	1703 (27.5%)	
3 ~ 4	9531 (25.6%)	7924 (25.5%)	1607 (25.9%)	
**Age at diagnosis, years**				<0.001
Mean (SD [Min-Max])	60.0 (12.5 [18.0-100])	59.4 (12.7 [18.0-100])	63.3 (10.7 [26.0-93.0])	
Median (IQR)	60.0 (52.0, 69.0)	60.0 (51.0, 68.0)	64.0 (56.0, 71.0)	
**Age group at diagnosis, years**				<0.001
(~44]	4193 (11.3%)	3908 (12.6%)	285 (4.6%)	
(45-59]	13487 (36.2%)	11590 (37.4%)	1897 (30.6%)	
(60-74]	14648 (39.4%)	11591 (37.4%)	3057 (49.3%)	
(75~)	4896 (13.2%)	3931 (12.7%)	965 (15.6%)	
**Race**				<0.001
White	31001 (83.3%)	25855 (83.3%)	5146 (82.9%)	
Black	4212 (11.3%)	3438 (11.1%)	774 (12.5%)	
Other	2011 (5.4%)	1727 (5.6%)	284 (4.6%)	
**Sex**				<0.001
Female	14050 (37.7%)	12123 (39.1%)	1927 (31.1%)	
Male	23174 (62.3%)	18897 (60.9%)	4277 (68.9%)	
**Grade**				0.026
Grade I	5179 (13.9%)	4317 (13.9%)	862 (13.9%)	
Grade II	20141 (54.1%)	16687 (53.8%)	3454 (55.7%)	
Grade III/IV	11904 (32.0%)	10016 (32.3%)	1888 (30.4%)	
**Tumor side**				0.501
Left	18188 (48.9%)	15132 (48.8%)	3056 (49.3%)	
Right	19036 (51.1%)	15888 (51.2%)	3148 (50.7%)	
**Histological type**				<0.001
ccRCC	22652 (60.9%)	18976 (61.2%)	3676 (59.3%)	
chRCC	1758 (4.7%)	1513 (4.9%)	245 (3.9%)	
paRCC	4294 (11.5%)	3414 (11.0%)	880 (14.2%)	
RCC (undefined)	6465 (17.4%)	5440 (17.5%)	1025 (16.5%)	
Other type RCC	2055 (5.5%)	1677 (5.4%)	378 (6.1%)	
**AJCC stage group**				<0.001
I	26326 (70.7%)	21790 (70.2%)	4536 (73.1%)	
II	4211 (11.3%)	3554 (11.5%)	657 (10.6%)	
III/IV	6687 (18.0%)	5676 (18.3%)	1011 (16.3%)	
**AJCC T stage**				<0.001
T1	26415 (71.0%)	21868 (70.5%)	4547 (73.3%)	
T2	4311 (11.6%)	3646 (11.8%)	665 (10.7%)	
T3/T4	6498 (17.5%)	5506 (17.7%)	992 (16.0%)	
**AJCC N stage**				<0.001
N0	36601 (98.3%)	30449 (98.2%)	6152 (99.2%)	
N1	623 (1.7%)	571 (1.8%)	52 (0.8%)	
**Surgery**				<0.001
Cryosurgery/Radiofrequency ablation	795 (2.1%)	595 (1.9%)	200 (3.2%)	
Nephrectomy	562 (1.5%)	463 (1.5%)	99 (1.6%)	
Partial nephrectomy	10863 (29.2%)	8986 (29.0%)	1877 (30.3%)	
Radical nephrectomy	25004 (67.2%)	20976 (67.6%)	4028 (64.9%)	
**Tumor size, mm**				
Mean (SD [Min-Max])	49.4 (28.9 [1.00-150])	49.7 (29.3 [1.00-150])	47.5 (27.1 [1.00-150])	
Median (IQR)	42.0 (28.0, 65.0)	42.0 (28.0, 65.0)	40.0 (28.0, 60.0)	
**Tumor size group**				<0.001
(~4] cm	18284 (49.1%)	15129 (48.8%)	3155 (50.9%)	
(4.1-7] cm	11487 (30.9%)	9503 (30.6%)	1984 (32.0%)	
(7.1-10] cm	5099 (13.7%)	4350 (14.0%)	749 (12.1%)	
(10~) cm	2354 (6.3%)	2038 (6.6%)	316 (5.1%)	
**SPM onset**				
None-SPM	31020 (83.3%)	31020 (100%)	/	/
With contralateral RCC SPM	747 (2.0%)	/	747 (12.0%)	
With ipsilateral RCC SPM	101 (0.3%)	/	101 (1.6%)	
With other-SPM	3703 (14.3%)	/	3703 (86.3%)	

SPM, second primary malignancy; AJCC, American Joint Committee on Cancer System; ccRCC, clear cell renal cell carcinoma; paRCC, papillary renal cell carcinoma; chRCC, chromophobe renal cell carcinoma; SD, standardized difference; IQR, interquartile range *. Matched by year of diagnosis and SEER register by propensity score matching at 1:5.

### Site Distribution of SPM and Time-to-SPM

**Figure 2:**
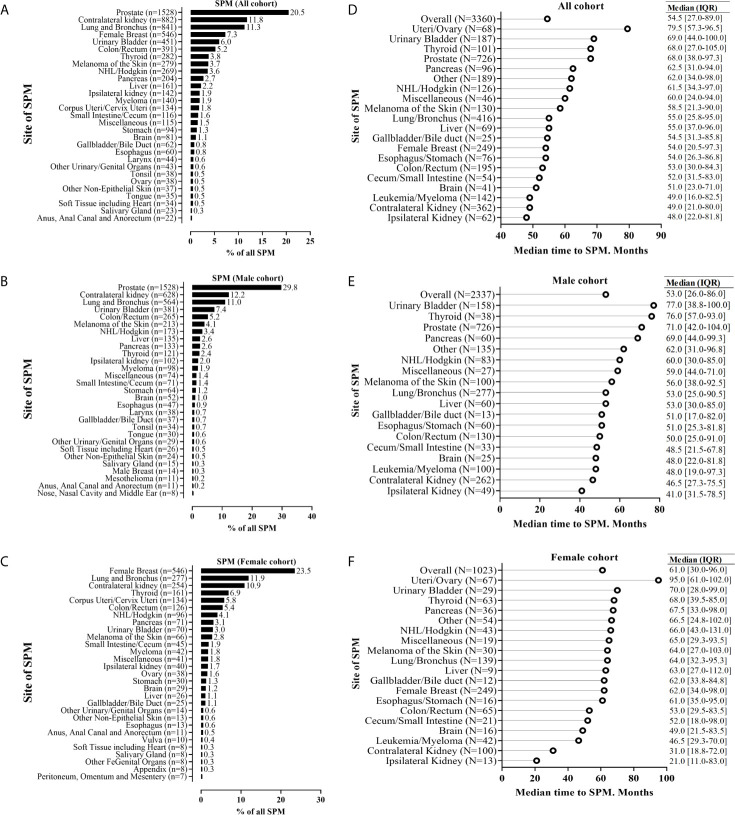
Anatomic distribution of second primary malignancy (SPM), and the time interval to SPM for patients who experienced SPM. The SPM distribution was stratified by the subgroup of all sexes **(A)**, male patients **(B)**, and female patients **(C)**; The time interval to SPM was stratified by the subgroup of all sexes **(D)**, male patients **(E)**, and female patients **(F)**. For the analysis of SPM distribution, we included crude data for 7461 patients with renal cell carcinoma by histologic confirmation, and just presented the top-29 sites of SPM. For the analysis of the time interval to SPM, we included patients with the first primary renal cell carcinoma diagnosed between 2004 and 2007 for >10-year follow-up and sample size >10.

**Figures 2A–C** shows the sites of SPM. Within the all-SPM cohort, the top-10 sites for SPM were the prostate gland (20.5%), contralateral kidney (11.8%), lungs and bronchi (11.3%), female breast (7.3%), bladder (6.0%), colon/rectum (5.2%), thyroid gland (3.8%), skin (melanoma) (3.7%), non-Hodgkin lymphoma/Hodgkin lymphoma (3.6%), and the pancreas (27%). Within sex subgroups, breast cancer (23.5%) and prostate cancer (29.8%) were the top sites for SPM in females and males, respectively. When considering different follow-up periods and race, the prostate gland, contralateral kidney, lung/bronchi, bladder, female breast, and colon/rectum were the common sites of SPM (**Figure 2** and **Supplementary Figures 1**, **2**).

The median time (in months) to SPM diagnosis was 54.5, 53.0, and 61.0, in all, male, and female cohorts, respectively (**Figures 2D–F**). Ipsilateral and contralateral kidneys had the shortest median time interval (in months) to SPM: 48.0 and 49.0, 41 and 46.5, and 21 and 31, in the entire cohort, male cohort, and female cohort, respectively.

**Figure 3:**
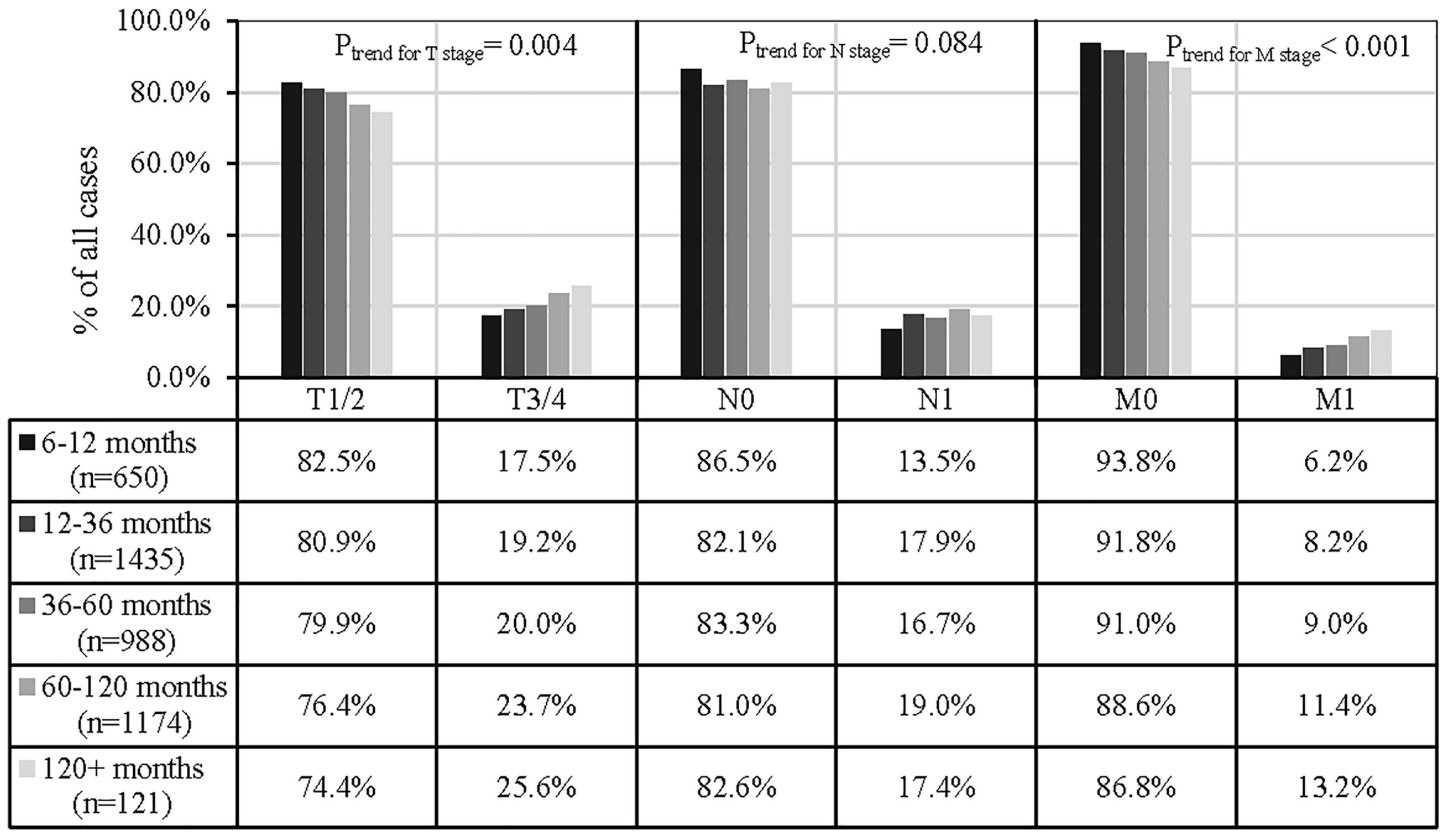
Associations between the time to SPM interval and SPM TNM stage. 1,836 SPMs were excluded from analyses due to a lack of information relating to the AJCC TNM stage or because the tumors were not solid).

Furthermore, **Figure 3** showed that a higher T (T3/4) and M (M1) stage SPM development was positively associated with a longer time-to-SPM interval (P_trend for T stage_= 0.004, and P_trend for T stage_< 0.001), but N stage of SPM, in the present analysis, did not observe significant association (P_trend for N stage_= 0.084).

We also observed 29.4% of clear-cell RCC, 40.1% of papillary RCC, and 60.0% of chromophobe RCC presenting with histologic changes compared with SPM of the kidney (**Supplementary Figure 3**). Concerning SPM in the contralateral kidney, 27.2%, 36.4%, and 59.6% of cases with clear-cell, papillary, and chromophobe RCC, respectively, presented histologic changes (**Supplementary Figure 4**). Histologic changes were also evident in cases with SPM in the ipsilateral kidney; such changes were observed in 41.2% of cases with clear-cell RCC, 73.3% of cases with papillary RCC, and 62.5% of cases with chromophobe RCC (**Supplementary Figure 5**).

### Age-, Time Interval-, and Site-Specific SIR for SPM

**Figure 4:**
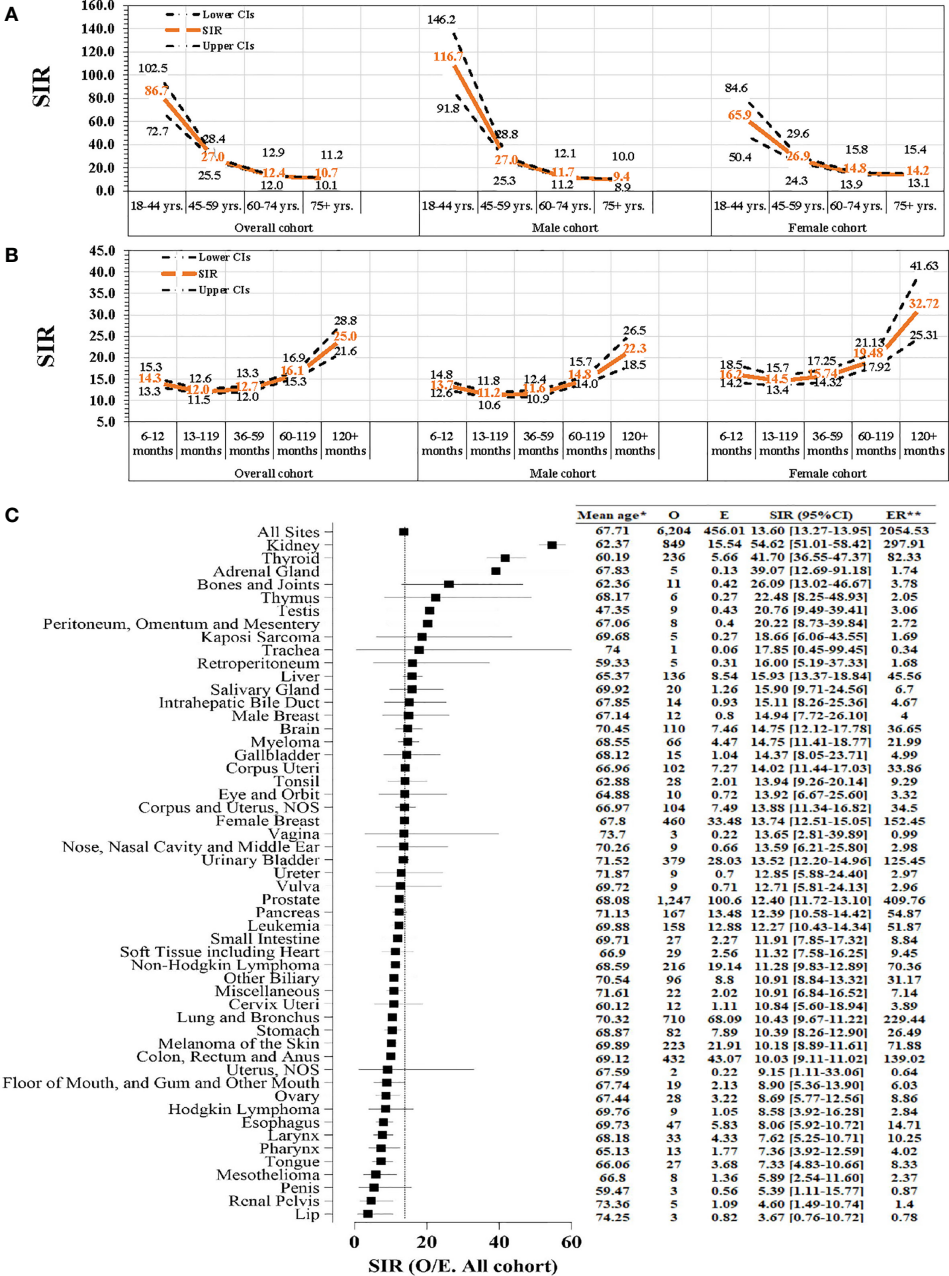
The standardized incidence ratio (SIR) of second primary malignancy (SPM). **(A)** Age-specific SIR for SPM was fitted for several age groups (18–44, 45–59, 60–74, and ≥75 years); **(B)** Time interval-specific SIR was based on several time-interval groups (<12, 12–35, 36–59, 60–119, and ≥120 months); **(C)** Site-specific SIR based on different sites of SPM. O, observed; E, expected; ER, excess risk. P < 0.05 (except SIR of site of lip). SIR was defined as the observed (O)-to-expected (E) ratio; *mean age at an event; **Excess risk is per 10,000.

Compared with the general population, younger RCC patients tended to have a significantly higher risk of developing SPM and presented a downwards trend for SPM incidence with increasing age (SIR_18–44-years_: 86.7; SIR_45–59-years_: 27.0; SIR_60–74-years_: 12.4; SIR_75+-years_: 10.7; P < 0.001 for all) (**Figure 4A**). An increased follow-up duration was associated with an increased incidence of SPM (SIR_12–35-months_: 12.0; SIR_36–59-months_: 12.7; SIR_60–19-months_: 16.1; SIR_120+-months_: 25.0; P < 0.001 for all) (**Figure 4B**). **Figure 4C** shows that the kidneys (SIR: 54.62; 95CI%: 51.01–58.42) had a significantly higher risk for SPM. **Supplementary Figure 6** and **Supplementary Figure 7** show similar results of site-specific SIR for SPM within female and male subgroups.

### Risk Factors for SPM Following a Diagnosis of Primary RCC

**Table 2** shows the results relating to risk factors for SPM onset. The risk of SPM was associated significantly with increasing age at the diagnosis of primary RCC (45–59 years *vs.* 18–44 years: sub-distribution hazard ratio [sHR] 2.13, 95%CI: 1.88–2.41, P < 0.001; 60–74 years *vs.* 18–44 years, sHR, 3.40, 95%CI: 3.01–3.85, P < 0.001; ≥75 *vs.* 18–44, sHR, 3.30, 95%CI: 2.88–3.78, P < 0.001). Moreover, black ethnicity, male sex, high family income, papillary RCC, low TNM stage, and cryosurgery/radiofrequency surgery (92% patients were T1a RCC, data not shown) were associated with a higher risk of SPM.

**Table 2:** Univariate and multivariate Fine and Gray competing risks regression of risk factors for second primary malignancy (SPM) among renal cell carcinoma patients.

	Nested Case-Control：All SPM vs. none-SPM		Nested Case-Control group 1：Non-RCC SPM vs. Non-SPM #		Nested Case-Control group 2：Contralateral RCC SPM vs. Non-SPM #		Nested Case-Control group 3 ¶：Ipsilateral RCC SPM vs. Non-SPM #	
	UnadjustedsHR (95%CI) *	AdjustedsHR (95%CI) **	Unadjusted sHR (95%CI) *	AdjustedsHR (95%CI) **	UnadjustedsHR (95%CI) *	AdjustedsHR (95%CI) **	UnadjustedsHR (95%CI) *	AdjustedsHR (95%CI) **
**Age at diagnosis, years ‡**								
(~44]	1 reference	1 reference	1 reference	1 reference	1 reference	1 reference	1 reference	1 reference
(45-59]	**2.12 (1.88-2.40)**	**2.13 (1.88-2.41)**	**2.72 (2.34-3.17)**	**2.71 (2.33-3.16)**	1.08 (0.86-1.36)	1.08 (0.86-1.37)	2.09 (0.90-4.85)	**2.46 (1.01-6.00)**
(60-74]	**3.32 (2.94-3.75)**	**3.40 (3.01-3.85)**	**4.61 (3.97-5.34)**	**4.69 (4.03-5.45)**	0.97 (0.77-1.22)	1.04 (0.82-1.33)	1.98 (0.84-4.67)	2.00 (0.82-4.91)
(75~)	**3.05 (2.67-3.48)**	**3.30 (2.88-3.78)**	**4.38 (3.74-5.13)**	**4.68 (3.98-5.50)**	**0.62 (0.44-0.87)**	0.79 (0.56-1.12)	2.44 (0.87-6.85)	2.49 (0.79-7.89)
**Race ‡**								
White	1 reference	1 reference	1 reference	1 reference	1 reference	1 reference	1 reference	1 reference
Black	**1.15 (1.07-1.25)**	**1.21 (1.12-1.31)**	**1.09 (1.00-1.18)**	**1.16 (1.06-1.27)**	**2.24 (1.88-2.66)**	**2.22 (1.84-2.67)**	0.96 (0.41-2.24)	1.24 (0.48-3.19)
Other	**0.86 (0.76-0.97)**	**0.86 (0.76-0.97)**	**0.85 (0.75-0.97)**	**0.87 (0.76-0.99)**	0.87 (0.60-1.25)	0.83 (0.57-1.21)	2.41 (0.97-5.97)	1.51 (0.59-3.83)
**Sex ‡**								
Female	1 reference	1 reference	1 reference	1 reference	1 reference	1 reference	1 reference	1 reference
Male	**1.39 (1.31-1.46)**	**1.41 (1.33-1.49)**	**1.35 (1.28-1.43)**	**1.40 (1.32-1.49)**	**1.52 (1.30-1.78)**	**1.34 (1.13-1.58)**	**1.71 (1.06-2.76)**	1.29 (0.77-2.17)
**Marital status ‡**								
Married	1 reference	1 reference	1 reference	1 reference	1 reference		1 reference	1 reference
Single/unmarried	**0.84 (0.78-0.91)**	0.96 (0.89-1.04)	**0.81 (0.75-0.88)**	0.97 (0.89-1.06)	1.10 (0.90-1.34)	0.97 (0.79-1.19)	0.53 (0.25-1.10)	0.80 (0.38-1.68)
Widowed/Divorced/Separated	0.95 (0.89-1.01)	0.96 (0.90-1.03)	0.99 (0.93-1.07)	0.99 (0.92-1.06)	**0.69 (0.56-0.85)**	**0.75 (0.60-0.94)**	0.70 (0.37-1.33)	0.71 (0.35-1.44)
**Population ‡**								
Counties	1 reference	1 reference	1 reference	1 reference	1 reference	1 reference	1 reference	1 reference
Rural	0.85 (0.68-1.05)	0.89 (0.71-1.10)	0.88 (0.70-1.10)	0.91 (0.72-1.14)	0.80 (0.42-1.52)	0.96 (0.49-1.85)	0.70 (0.10-4.79)	0.66 (0.12-3.56)
Urban	0.99 (0.92-1.07)	1.02 (0.94-1.11)	0.98 (0.90-1.07)	1.00 (0.92-1.10)	0.90 (0.71-1.15)	1.05 (0.81-1.36)	1.45 (0.76-2.77)	1.35 (0.69-2.63)
**Region ‡**								
East	1 reference	1 reference	1 reference	1 reference	1 reference	1 reference	1 reference	1 reference
Northern Plains	0.99 (0.91-1.08)	0.96 (0.88-1.05)	1.01 (0.92-1.10)	0.96 (0.87-1.06)	0.97 (0.77-1.21)	0.95 (0.75-1.21)	1.11 (0.54-2.26)	0.79 (0.36-1.76)
Pacific Coast	0.99 (0.94-1.04)	1.01 (0.95-1.07)	1.00 (0.94-1.06)	1.01 (0.95-1.07)	0.99 (0.85-1.16)	1.12 (0.95-1.32)	1.08 (0.68-1.71)	0.93 (0.55-1.57)
Southwest	1.01 (0.87-1.17)	1.07 (0.92-1.24)	1.02 (0.87-1.19)	1.05 (0.90-1.23)	0.93 (0.57-1.53)	1.12 (0.68-1.84)	1.03 (0.25-4.31)	0.84 (0.15-4.57)
**The median family income quartile ‡**								
1 or less	1 reference	1 reference	1 reference	1 reference	1 reference	1 reference	1 reference	1 reference
1 ~ 2	1.05 (0.97-1.13)	1.05 (0.98-1.13)	1.05 (0.97-1.13)	1.06 (0.98-1.15)	0.98 (0.79-1.21)	0.96 (0.77-1.20)	1.42 (0.76-2.66)	1.38 (0.71-2.65)
2 ~ 3	**1.10 (1.03-1.19)**	**1.11 (1.03-1.20)**	**1.10 (1.02-1.19)**	**1.11 (1.03-1.21)**	1.09 (0.89-1.34)	1.14 (0.92-1.41)	1.23 (0.65-2.34)	1.22 (0.64-2.35)
3 ~ 4	1.07 (0.99-1.15)	**1.09 (1.00-1.18)**	1.07 (0.99-1.16)	**1.09 (1.00-1.19)**	1.07 (0.87-1.32)	1.16 (0.92-1.46)	0.89 (0.45-1.77)	1.27 (0.60-2.72)
**Grade ‡**								
Grade I	1 reference	1 reference	1 reference	1 reference	1 reference	1 reference	1 reference	1 reference
Grade II	**1.07 (1.00-1.16)**	1.05 (0.97-1.13)	**1.10 (1.01-1.19)**	1.06 (0.98-1.15)	1.13 (0.90-1.41)	1.12 (0.90-1.40)	**1.97 (1.08-3.60)**	1.47 (0.76-2.85)
Grade III/IV	1.00 (0.93-1.09)	0.98 (0.91-1.07)	1.02 (0.94-1.12)	0.98 (0.90-1.07)	**1.28 (1.01-1.62)**	**1.34 (1.06-1.71)**	1.54 (0.69-3.45)	1.43 (0.61-3.33)
**Histological type ‡**								
ccRCC	1 reference	1 reference	1 reference	1 reference	1 reference	1 reference	1 reference	1 reference
chRCC	**0.82 (0.72-0.94)**	**0.85 (0.74-0.96)**	**0.83 (0.72-0.95)**	**0.86 (0.75-0.99)**	0.75 (0.50-1.12)	**0.62 (0.41-0.94)**	0.52 (0.12-2.17)	0.86 (0.19-3.82)
paRCC	**1.29 (1.20-1.39)**	**1.10 (1.02-1.19)**	**1.23 (1.13-1.33)**	1.05 (0.97-1.14)	**1.81 (1.51-2.19)**	**1.33 (1.09-1.62)**	0.65 (0.32-1.33)	0.60 (0.28-1.30)
RCC (undefined)	**0.89 (0.83-0.96)**	**0.87 (0.81-0.94)**	**0.90 (0.83-0.97)**	**0.88 (0.82-0.95)**	0.88 (0.72-1.09)	0.83 (0.67-1.03)	**0.50 (0.26-0.99)**	**0.46 (0.22-0.96)**
Other type RCC	1.10 (0.99-1.23)	1.08 (0.97-1.20)	**1.12 (1.00-1.26)**	1.10 (0.98-1.23)	1.02 (0.73-1.41)	0.94 (0.68-1.29)	0.99 (0.39-2.52)	1.77 (0.66-4.76)
**AJCC TNM stage group ‡**								
I	1 reference	1 reference	1 reference	1 reference	1 reference	1 reference		
II	**0.88 (0.81-0.95)**	0.96 (0.88-1.05)	**0.90 (0.82-0.98)**	0.96 (0.88-1.05)	0.91 (0.72-1.15)	0.98 (0.76-1.25)		
III/IV	**0.87 (0.81-0.93)**	**0.86 (0.80-0.93)**	**0.88 (0.82-0.94)**	**0.84 (0.77-0.91)**	0.84 (0.69-1.01)	0.92 (0.74-1.13)		
**Tumor size of first primary RCC θ**								
(~4] cm	1 reference	1 reference	1 reference	1 reference	1 reference	1 reference		
(4.1-7] cm	0.99 (0.94-1.05)	1.04 (0.98-1.10)	1.02 (0.96-1.08)	1.03 (0.96-1.10)	0.99 (0.84-1.17)	1.14 (0.95-1.37)		
(7.1-10] cm	**0.83 (0.76-0.89)**	**0.91 (0.83-0.99)**	**0.82 (0.76-0.90)**	**0.87 (0.79-0.95)**	0.96 (0.78-1.19)	1.19 (0.93-1.52)		
(10~) cm	**0.75 (0.67-0.84)**	**0.85 (0.75-0.96)**	**0.75 (0.66-0.86)**	**0.82 (0.71-0.93)**	1.09 (0.81-1.46)	1.30 (0.93-1.80)		
**AJCC T stage §**								
T1	1 reference	1 reference	1 reference	1 reference	1 reference	1 reference		
T2	**0.87 (0.80-0.94)**	0.96 (0.88-1.04)	**0.89 (0.82-0.97)**	0.96 (0.87-1.05)	0.90 (0.72-1.13)	0.97 (0.76-1.25)		
T3/T4	**0.88 (0.82-0.94)**	**0.89 (0.83-0.96)**	**0.89 (0.83-0.96)**	**0.87 (0.81-0.95)**	0.85 (0.70-1.03)	0.99 (0.80-1.22)		
**AJCC N stage §**								
N0	1 reference	1 reference	1 reference	1 reference	1 reference	1 reference		
N1	**0.47 (0.36-0.62)**	**0.51 (0.39-0.68)**	**0.47 (0.35-0.63)**	**0.50 (0.37-0.67)**	**0.34 (0.14-0.81)**	**0.32 (0.13-0.79)**		
**Surgery for first primary RCC ‡**								
Cryosurgery/Radiofrequency	1 reference	1 reference	1 reference	1 reference	1 reference	1 reference	1 reference	1 reference
Nephrectomy	**0.59 (0.46-0.75)**	**0.71 (0.56-0.90)**	**0.68 (0.53-0.87)**	0.82 (0.64-1.06)	0.63 (0.24-1.62)	0.55 (0.21-1.45)		
Partial nephrectomy	**0.67 (0.58-0.77)**	**0.78 (0.67-0.90)**	**0.67 (0.57-0.79)**	**0.80 (0.68-0.94)**	1.59 (0.92-2.75)	1.45 (0.84-2.52)	**0.41 (0.25-0.70)**	**0.30 (0.17-0.55)**
Radical nephrectomy	**0.56 (0.48-0.64)**	**0.64 (0.56-0.74)**	**0.60 (0.51-0.70)**	**0.70 (0.60-0.82)**	1.08 (0.63-1.86)	1.00 (0.58-1.72)		

SPM, second primary malignancy; CI, confidence interval; sHR, Sub-distribution hazard ratio; AJCC, American Joint Committee on Cancer system; RCC, renal cell carcinoma; T, Tumor; N, lymph node; ccRCC, clear cell renal cell carcinoma; paRCC, papillary renal cell carcinoma; chRCC, chromophobe renal cell carcinoma. Bold type indicates that the P-value is significant (P< 0.05). *Univariate Fine and Grey proportional risk regression analysis. **Multivariate Fine and Grey proportional risk regression analysis. ^#^Each comparison group was matched with 1:5 propensity score, the matched data was shown in the **Supplementary Table 2**. ^¶^First primary renal cell carcinoma with the AJCC stage of T1aN0M0 (number of cases with SPM in ipsilateral renal cell carcinoma vs. control group was 82 vs. 410) was included. ^ǂ^Results of multivariate Fine and Grey proportional risk regression analysis with all variables adjusted. ^θ^Results of multivariate Fine and Grey proportional risk regression analysis with adjusting for the AJCC N stage and all variables of ǂ except the AJCC stage group. ^§^Results of multivariate Fine and Grey proportional risk regression analysis with adjusting for all variables of ǂ except AJCC stage group and tumor size group.

### Survival and Risk Predictors Following the Diagnosis of Primary RCC and SPM

**Figure 5:**
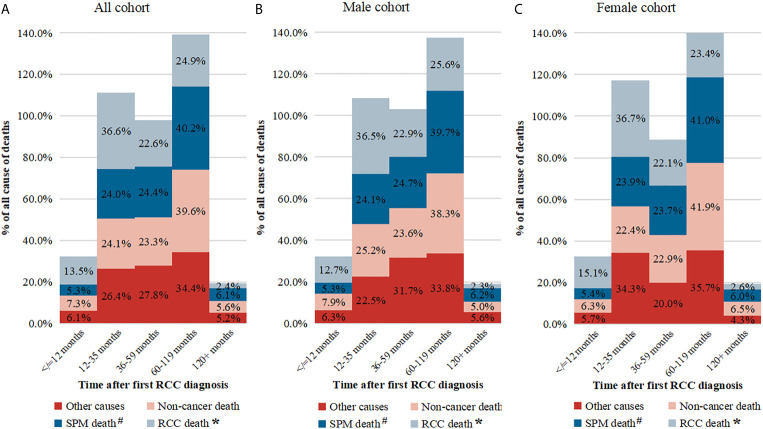
The proportion of death causes at different time intervals. **(A)** Total cohort; **(B)** Male cohort; **(C)** Female cohort. # Not included patients of second primary malignant (SPM) of kidney; * Included cases of SPM of kidney.

**Figure 6:**
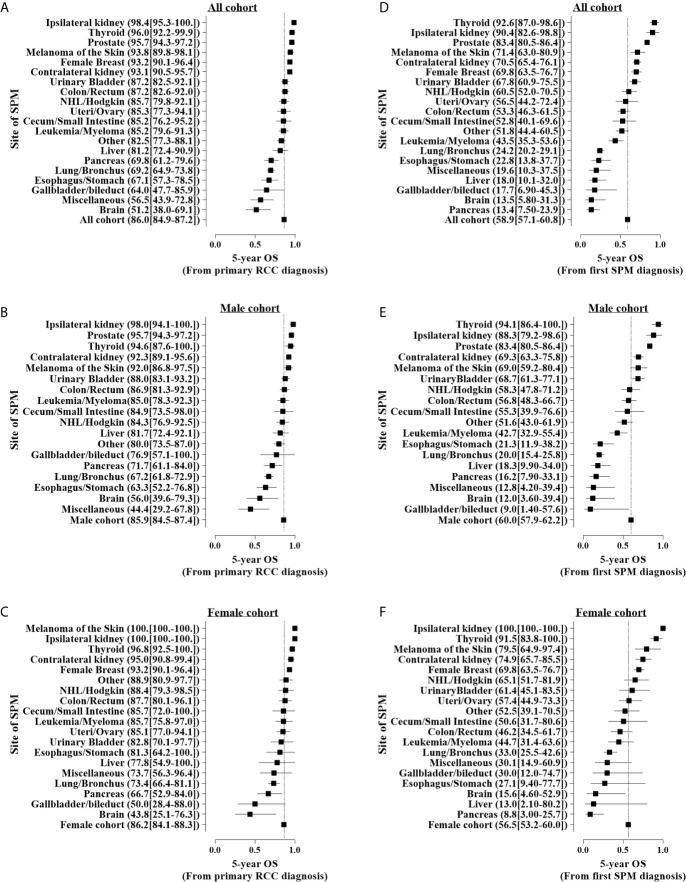
Five-year overall survival (OS) for patients with different sites of second primary malignancy (SPM) onset. **(A–C)** Five-year OS from the first primary renal cell carcinoma stratified by different sex and **(D–F)** 5-year OS after SPM onset. Patients diagnosed in 2004–2007 were used for survival analyses with the Kaplan–Meier method.

At the final follow-up of crude median 76 months, 7.3% and 4.7% of patients had died from RCC and other cancers, respectively (results from unmatched data; data not shown). Compared with non-SPM patients, those with SPM were associated with worse OS (HR: 1.26; 95%CI: 1.20–1.32; P < 0.001) (**Table 3**). The proportion of deaths from SPM exceeds that of deaths from RCC 3 years after the first RCC diagnosis (**Figure 5**). Survival analyses showed that 5-year OS for T1-4N0-1M0 RCC with SPM was 85.9% from the diagnosis of primary RCC (**Figure 6A**) and 58.9% from the SPM diagnosis (**Figure 6D**). SPM of the pancreas, brain, gallbladder/bile duct, liver, miscellaneous tissues, esophagus/stomach, and lungs/bronchi was associated with a poor survival outcome, with 5-year OS of 13.4%, 13.5%, 17.7%, 18.0%, 19.6%, 22.8%, and 24.2% from the SPM diagnosis, respectively. Similar results were obtained for female and male cohorts (**Figures 6B, C, E, F**).

**Table 3:** Risk predictors of all-cause mortality by the time-to-SPM and site of SPM.

	Survival function from the first primary RCC diagnosis				Survival function from the SPM diagnosis			
	Unadjusted sHR (95%CI)	*P-*value	Adjusted (s) sHR (95%CI)	*P-*value	Unadjusted sHR (95%CI)	*P-*value	Adjusted sHR (95%CI)	*P-*value
Time to SPM *§								
>120 months	1 reference		1 reference		1 reference		1 reference	
6<time≤12 months	5.74 (3.87-8.49)	<0.001	27.56 (18.27-41.56)	<0.001	0.60 (0.40-0.89)	0.011	0.86 (0.57-1.29)	0.470
12<time≤24 months	5.32 (3.60-7.85)	<0.001	25.20 (16.77-37.86)	<0.001	0.65 (0.44-0.97)	0.033	0.94 (0.63-1.41)	0.767
24<time≤36 months	4.89 (3.30-7.24)	<0.001	18.65 (12.39-28.06)	<0.001	0.72 (0.48-1.08)	0.112	0.99 (0.66-1.49)	0.962
36<time≤48 months	3.54 (2.38-5.28)	<0.001	12.16 (8.04-18.38)	<0.001	0.65 (0.44-0.98)	0.040	0.92 (0.61-1.39)	0.696
48<time≤60 months	3.48 (2.33-5.20)	<0.001	9.90 (6.55-14.96)	<0.001	0.79 (0.52-1.18)	0.253	1.05 (0.69-1.59)	0.821
60<time≤72 months	2.96 (1.96-4.45)	<0.001	7.34 (4.82-11.16)	<0.001	0.82 (0.54-1.24)	0.338	1.05 (0.69-1.60)	0.820
72<time≤84 months	2.66 (1.76-4.04)	<0.001	5.03 (3.28-7.70)	<0.001	0.94 (0.61-1.43)	0.757	1.11 (0.73-1.71)	0.625
84<time≤96 months	2.30 (1.50-3.53)	<0.001	3.99 (2.59-6.17)	<0.001	0.97 (0.63-1.49)	0.880	1.06 (0.69-1.65)	0.784
96<time≤108 months	1.78 (1.12-2.84)	0.015	2.81 (1.75-4.52)	<0.001	0.97 (0.60-1.55)	0.898	1.18 (0.73-1.90)	0.493
108<time≤120 months	1.34 (0.80-2.26)	0.269	2.01 (1.18-3.40)	0.010	0.85 (0.50-1.45)	0.559	0.93 (0.54-1.60)	0.786
Types of SPM *#								
Ipsilateral kidney	1 reference		1 reference		1 reference		1 reference	
Contralateral kidney	2.34 (1.23-4.45)	0.009	1.43 (0.74-2.76)	0.286	1.63 (0.86-3.10)	0.133	1.53 (0.79-2.95)	0.206
Brain	15.68 (7.97-30.85)	<0.001	26.36 (12.05-57.65)	<0.001	21.06 (10.68-41.52)	<0.001	31.40 (14.24-69.23)	<0.001
Cecum/Small Intestine	4.81 (2.40-9.63)	<0.001	1.56 (0.76-3.21)	0.223	3.67 (1.83-7.36)	<0.001	1.95 (0.95-3.99)	0.068
Colon/Rectum	5.07 (2.67-9.63)	<0.001	2.24 (1.16-4.31)	0.016	3.84 (2.02-7.30)	<0.001	2.45 (1.27-4.72)	0.008
Esophagus/Stomach	10.23 (5.30-19.74)	<0.001	3.46 (1.76-6.79)	<0.001	9.37 (4.86-18.07)	<0.001	3.28 (1.67-6.44)	0.001
Female Breast	2.12 (1.10-4.07)	0.024	1.51 (0.77-2.98)	0.234	1.64 (0.85-3.16)	0.136	1.54 (0.78-3.03)	0.214
Gallbladder/bile duct	13.16 (6.53-26.53)	<0.001	4.95 (2.40-10.20)	<0.001	11.69 (5.80-23.55)	<0.001	6.12 (2.97-12.60)	<0.001
Liver	9.30 (4.82-17.95)	<0.001	5.18 (2.64-10.17)	<0.001	10.01 (5.19-19.32)	<0.001	5.94 (3.03-11.66)	<0.001
Lung/Bronchus	9.85 (5.26-18.44)	<0.001	3.03 (1.59-5.78)	0.001	8.60 (4.59-16.09)	<0.001	3.03 (1.59-5.78)	0.001
Pancreas	12.11 (6.35-23.09)	<0.001	4.46 (2.29-8.68)	<0.001	15.06 (7.89-28.72)	<0.001	5.85 (3.00-11.42)	<0.001
Prostate	1.74 (0.92-3.29)	0.087	0.43 (0.22-0.83)	0.011	1.21 (0.64-2.28)	0.556	0.49 (0.26-0.95)	0.035
Thyroid	1.20 (0.57-2.52)	0.635	0.76 (0.36-1.61)	0.468	0.80 (0.38-1.68)	0.552	0.84 (0.40-1.80)	0.658
Urinary Bladder	3.63 (1.90-6.94)	<0.001	3.65 (1.84-7.26)	<0.001	2.52 (1.32-4.82)	0.005	3.91 (1.96-7.79)	<0.001
Uteri/Ovary	3.46 (1.73-6.91)	<0.001	2.03 (0.99-4.16)	0.053	2.96 (1.48-5.92)	0.002	2.15 (1.05-4.40)	0.036
Types of SPM **								
All site of SPM (None-SPM as a reference) ¶	1.40 (1.33-1.46)	<0.001	1.26 (1.20-1.32)	<0.001	–	–	–	–
All site of SPM (None-SPM as a reference) θ	1.34 (1.28-1.42)	<0.001	1.23 (1.17-1.30)	<0.001	–	–	–	–

SPM, second primary malignancy; CI, confidence interval; sHR, Sub-distribution hazard ratio; AJCC, American Joint Committee on Cancer system; RCC, renal cell carcinoma.

* All SPM in solid malignancy (n= 5008) exclude acute/chronic leukemia/myeloma, melanoma of the skin, NHL/Hodgkin, miscellaneous.

§ Multivariate analysis with all other covariables (age of first primary RCC [for OS from first primary RCC diagnosis analysis] or the age of SPM [for OS from SPM diagnosis analysis], respectively], sex, race, marital status, population, region, family income, tumor grade, histological type of first primary RCC, tumor size of first primary RCC, tumor TNM stage of RCC and SPM, and treatment of RCC and SPM, and types of SPM).

# Multivariate analysis with all other covariables (age of first primary RCC [for OS from first primary RCC diagnosis analysis] or the age of SPM [for OS from SPM diagnosis analysis], respectively], sex, race, marital status, population, region, family income, tumor grade, histological type of first primary RCC, tumor size of first primary RCC, tumor TNM stage of RCC and SPM, and treatment of RCC and SPM, and time-to-SPM).

** Multivariate analysis with all other covariables (age of first primary RCC, sex, race, marital status, population, region, family income, tumor grade, histological type of first primary RCC, tumor TNM stage, and tumor size of first primary RCC) based the data of all cohort including the patient with none-SPM and SPM.

¶ Univariate and multivariate Cox proportional risk regression before propensity score matching.

θ Univariate and multivariate Cox proportional risk regression analysis after 1: 5 propensity score matching.

Because the time interval to the diagnosis of SPM and the location of SPM may have an impact on the prognosis, we further adjusted other covariables in the multivariate COX model to analyze the impact of the two on the prognosis (**Table 3**). We found that an early SPM diagnosis associated with a lower OS (6< time ≤12 months *vs.* >120 months; HR: 5.75; 95% CI: 3.87–8.49; P<0.001), however, it was not associated with the OS from the SPM onset. Compared with SPM in the ipsilateral kidney, those with SPM in the contralateral kidney did not show significantly worse OS since the first RCC diagnosis or from the SPM diagnosis. **Table 4** shows that a higher SPM tumor stage, with no surgical treatment, reduced OS significantly.

**Table 4:** Risk predictors of all-cause mortality after SPM diagnosis by the TNM stage or stage groups of SPMs and surgical treatment among all patients with solid SPM*.

	Unadjusted HR (95%CI) §	*P*-value	Adjusted HR (95%CI)#	*P*-value
**Invasion of SPM ǂ**				
Local	1 reference		1 reference	
Regional	2.19 (1.92-2.50)	<0.001	1.59 (1.37-1.84)	<0.001
Distant	9.13 (8.06-10.33)	<0.001	3.83 (3.28-4.47)	<0.001
**AJCC stage group of SPM ǂ**				
I	1 reference		1 reference	
II	0.89 (0.77-1.04)	0.129	1.32 (1.10-1.59)	0.003
III	2.55 (2.16-3.00)	<0.001	2.02 (1.70-2.41)	<0.001
IV	7.03 (6.10-8.11)	<0.001	3.95 (3.34-4.67)	<0.001
**AJCC T stage of SPM** ¶				
T1	1 reference		1 reference	
T2	1.46 (1.27-1.68)	<0.001	1.45 (1.25-1.68)	<0.001
T3/T4	3.44 (3.02-3.91)	<0.001	1.66 (1.42-1.93)	<0.001
Ta/Tis	0.91 (0.70-1.19)	0.500	0.42 (0.29-0.60)	<0.001
**AJCC N stage of SPM** ¶				
N0	1 reference		1 reference	
N1	2.22 (1.91-2.60)	<0.001	1.15 (0.97-1.38)	0.112
N2	5.37 (4.57-6.30)	<0.001	1.61 (1.33-1.95)	<0.001
N3	8.19 (6.20-10.83)	<0.001	2.06 (1.51-2.80)	<0.001
**AJCC N stage of SPM** ¶				
M0	1 reference		1 reference	
M1	7.50 (6.66-8.45)	<0.001	2.41 (2.09-2.79)	<0.001
**Surgical treatment for SPM**				
NO	1 reference		1 reference	
Yes	0.35 (0.32-0.39)	<0.001	0.40 (0.35-0.46)	<0.001

SPM, second primary malignancy; CI, confidence interval; AJCC, American Joint Committee on Cancer system; RCC, renal cell carcinoma; T, Tumor; N, lymph node; M, Metastasis.

*All SPM in solid malignancy (n= 5008) exclude acute/chronic leukemia/myeloma, melanoma of the skin, NHL/Hodgkin, miscellaneous.

§ Univariate Cox proportional risk regression analysis.

#Multivariate analysis with covariables adjusted (age at the time of diagnosis of SPM, sex, race, marital status, population, region, family income, tumor grade, histological type of first primary RCC, tumor size of first primary RCC, tumor TNM stage [or stage groups], time-to-SPM, types of SPM, and treatment of RCC and SPM).

ǂ Invasion of SPM and AJCC TNM stage groups were separately included in the multivariate Cox proportional risk regression with other covariables.

¶ AJCC T, N, M were together included in the Cox proportional risk regression.

## Discussion

Although the long-term cancer-special survival of RCC was good (4, 14), for some patients, this benefit was reduced by an SPM onset since it threatened long-term OS (7, 8). Besides, the present study showed that RCC patients seem to be at a higher risk of SPM onset when compared to the general population. Therefore, it’s of great importance to perform long-term surveillance to detect not only local/distant recurrence but also an early SPM onset. The three most important guidelines (National Comprehensive Cancer Network, European Association of Urology, and American Urological Association) for the surveillance of RCC patients following surgery differ in terms of their recommendations for imaging modalities and the frequency of imaging (15). Although there is no consensus concerning an optimal surveillance strategy for patients after RCC treatment, it is often recommended that patients are followed up closely for 3–5 years after surgery (16, 17). Tumor recurrence and complications must be considered for prognosis prediction, and most occur <5 years after treatment (18), this might lead some RCC survivors to consider ending surveillance after this interval. Although a prior study observed that increasing the imaging frequency during follow-up does not improve survival for RCC patients with recurrence (19), it does not mean that long-term follow-up is unnecessary, and to some extent, SPM can be detected early. In the present study, we found that the median time-to-SPM interval was 54.5 for RCC patients with SPM, and most occurred 3 years and more after RCC treatment, which consistent with the prior report (20). Furthermore, the SIR of SPM increased by follow-up duration, and longer time duration from primary RCC diagnosis to SPM diagnosis was associated with the worse stage (T3/4, M1) of SPM in the RCC patients with SPM onset, which significantly threaten the OS from the diagnosis of SPM. One reason for this result may be a delayed diagnosis of SPM.

In the present study, we identified a series of independent high-risk predictors for SPM: increased age at the diagnosis of primary RCC, black ethnicity, male sex, patients with a high family income, papillary types of cancer, small tumors, absence of lymph-node invasion, a lower T stage, and a lower American Joint Committee on Cancer TNM stage. Our results further suggest that although some patients, such as those with a low TNM stage of RCC, may represent a low-risk group for recurrence after RCC (21), further follow-up is necessary because this group has a risk of developing SPM. Furthermore, SPM emergence is related to sex and the pathological type of RCC, thereby indicating that SPM may be related to certain genes or other types of environmental exposure. Interestingly, SPM is related to economic factors and may be more prevalent in patients with a certain financial status. Some patients may pay more attention to their follow-up and general health status and also have the financial means to afford the costs associated with follow-up (22).

For predicting non-RCC SPM, the risk of SPM increases with age. Age is an independent risk factor for cancer (23) because the risk of developing tumors increases with age. As long as patients survive long enough, they are likely to develop more than two types of malignant tumors in their lives. It has been reported that 33% of cancer survivors aged >60 years will be diagnosed with another type of cancer (24). For some patients, RCC is only one of the earliest malignant tumors that could develop; however, with improvements in the prognosis and survival of RCC patients, it is now possible to detect SPM during follow-up. In elderly patients with RCC as their first cancer, SPM may be recorded shortly after the RCC diagnosis. In contrast, younger patients may need a longer follow-up duration for SPM to be detected (perhaps because they need to reach a certain age for successful detection). Therefore, it is possible that SPM was not detected by the end of follow-up in our study, or that various competitive events prevented SPM.

An interesting result was that cryosurgery/radiofrequency surgery was associated with a higher risk of SPM. After furtherly analyzing our data, we found that about 92% of RCC patients who underwent cryosurgery/radiofrequency surgery were T1a stage, and with a median tumor size of 2.5cm (data not shown). Prior studies indicated that cryosurgery/radiofrequency surgery in T1a RCC patients showed a comparable survival outcome in comparison with PN (25, 26), especially for small tumor size RCC (27). Therefore, it may be linked to the use of these treatments only in low TNM stage tumors, which are associated with a higher risk of SPM. Pecoraro, A et al. (28) found that cryoablation versus PN predisposes to higher cancer-specific mortality in patients with nonmetastatic pathological T1b RCC. This may be due to a higher risk of recurrence linked to these treatments. Therefore, it is important to exclude those misclassifications of some early recurrence as ipsilateral SPM. However, this risk may be not completely be ruled out as access to the clinical data is limited.

In addition, for assessment of SPM risk, we must consider genetic and family history, cancer treatment, as well as environmental and lifestyle factors (e.g., smoking, excessive drinking, excessive eating) (7). Cancer survivors with a family history of cancer may have a higher-than-average risk of SPM. Individuals with a family history of cancer syndromes could be candidates for genetic testing and should be recommended for genetic counseling (29, 30). The most common cancer syndromes identified include hereditary breast cancer, ovarian cancer, and hereditary non-polyposis colorectal cancer (Lynch syndrome) (7). Mutations in breast cancer type 1 susceptibility protein (BRCA1) and BRCA2 genes are associated with a high risk of SPM for breast cancer or ovarian cancer. Mutations in mismatch repair genes increase the risk of various cancers, including gastrointestinal and gynecological cancers (7, 31). Prior research suggests that SPM can be attributed mainly to previous cancer radiotherapy or chemotherapy (32–35). Although, the RCC of common histology is not sensitive to radiotherapy or chemotherapy, so they are not recommended as a treatment for patients with RCC (36), radiotherapy has a central role in the treatment of many adult cancers, but it also increases the SPM risk caused by radiotherapy. For example, patients with cancer of the prostate gland have an increased risk of colorectal cancer and bladder cancer after local radiotherapy to the pelvis (10). Patients with testicular cancer receiving radiotherapy to the mediastinum, abdominal aorta, and pelvis can have an increased risk of SPM in the lungs, thyroid gland, esophagus, and stomach (7, 33). Similarly, chemotherapy can increase the risk of SPM. In addition to increasing the risk of acute leukemia, alkylating chemotherapy is also associated with several solid tumors, especially lung cancer, gastrointestinal cancer, sarcoma, and bladder cancer (7). Importantly, the relationship between radiotherapy and chemotherapy for pre-SPM is, to a large extent, dose-dependent, but the variability of the effect indicates an important relationship with genetic susceptibility (7).

The kidney showed the highest SIR. The contralateral kidney was a common site of SPM cases in RCC survivors. Early detection of small, local SPM in the contralateral kidney would benefit from partial nephrectomy (PN), particularly in patients who experienced radical nephrectomy (RN) for their first primary RCC. Conversely, considering the risk of cancer in the contralateral kidney following surgery for primary RCC, PN is recommended for the first kidney surgery to retain renal function, even if contralateral SPM occurs. Early detection of a local small RCC is critical and could benefit from PN, and this method may protect normal renal parenchyma (37–40). Additionally, healthy persons or RCC survivors should be screened for malignancies associated with the prostate gland, breast, lung, bladder, colon/rectum, and thyroid gland (41); these types of tumors accounted for most of the SPM cases seen in the present study. Our results indicated that early occurrence of SPM could exert an impact on OS, but survival could be prolonged if new cases of SPM were detected and treated early. Moreover, if SPM cases were detected later, or involved a higher stage of solid SPM, then curative surgical treatment would be difficult and threaten long-term OS.

Different sites of SPM were associated significantly with outcomes. For example, SPM of the thyroid gland, ipsilateral RCC, and prostate cancer all showed excellent 5-year OS after the SPM diagnosis. After adjustment for other risk factors, we found that SPM of contralateral RCC, cecum/small intestine, female breast, thyroid gland, and prostate gland had similar or significantly better OS when compared with that for ipsilateral RCC. Worse OS was associated with SPM of the brain, liver, gallbladder/bile duct, lungs/bronchi, pancreas, and esophagus/stomach; furthermore, these sites of SPM also had a higher SIR compared with that in the general population. Although the prognosis of different types of tumors is different, a prior study had observed that a prior cancer history could impact the OS of patients newly diagnosed with cancer (20), it found that colon and rectum, bone and soft tissues, melanoma, breast, cervix uteri, corpus and uterus, prostate, urinary bladder, kidney, and renal pelvis, eye and orbits, thyroid, had inferior OS than those patients without prior cancer history; but cancers of nasopharynx, esophagus, stomach, liver, gallbladder, pancreas, lung, ovary and brain showed a similar OS between patients with/without prior cancer history (20).

Our study had four main limitations. First, analyses were carried out using a registry-based dataset with inherent limitations. For example, we did not have access to detailed clinical information (e.g., comorbidities or poor performance) that led to death within a period of follow-up. This represents a vital competing risk for SPM and could not be adjusted in our multivariate analysis for SPM prediction. In addition, when analyzing the prediction of SPM for ipsilateral and contralateral RCC, we did not have access to information relating to hereditary RCCs, such as von Hippel–Lindau, hereditary papillary renal carcinoma, Birt–Hogg–Dubé syndrome, or hereditary leiomyomatosis and RCC (42); these factors have been identified as significant predictors for the metachronous *de novo* development of RCC over long-term follow-up. We did not have access to information relating to environmental exposure, lifestyle, family history, or genetic mutations, all of which are risk factors for SPM. Second, patients with primary RCC or other diseases would, in general, pay more attention to routine cancer screening or surveillance than the general population, thereby increasing the chances of identifying SPM in RCC survivors. Therefore, surveillance bias may have been present in our study. Third, after the diagnosis of the first primary RCC, the preexisting or concomitant malignancy, metastatic diseases, or relapse in patients with RCC may have confounded the subsequent detection of SPM. To control for confounding factors, we included only patients with SPM who had been diagnosed ≥6 months after the diagnosis of the first primary RCC as our study cohort. When using the SEER database, we maintained quality assurance by undertaking systematic and standardized procedures for data collection. Fourth, due to a lack of information relating to local recurrence, we included patients with ipsilateral RCC; these cases may have been recurrences rather than SPM. However, >90% of ipsilateral RCCs were diagnosed >3 years after the RCC diagnosis; 50% were diagnosed after >5 years. Furthermore, 35.6% of patients with RCC showed histologic changes between the first and second occurrence of ipsilateral RCC.

## Conclusions

Our analyses demonstrated a higher incidence of SPM among RCC patients surviving over the long term when compared with that in the general population. Age at the diagnosis of the first primary RCC, ethnicity, sex, economic status, histologic type of RCC, and tumor stage was associated significantly with SPM. The stage and site of SPM showed particularly strong associations with OS. Lifetime follow-up and cancer screening should be recommended for RCC survivors. SPM can threaten long-term survival, but patients with low-grade/early-stage SPM could benefit from aggressive surgical treatment for solid tumors. According to the patient’s age and time interval after the RCC diagnosis, monitoring high-risk RCC patients by site- and time-specific surveillance strategies are worthwhile.

## Data Availability Statement

Publicly available datasets were analyzed in this study. This data can be found here: https://seer.cancer.gov.

## Ethics Statement

The ethics approval was waived by the Ethics Review Board of Tongji Hospital within Tongji Medical College (Huazhong University of Science and Technology, Wuhan, China).

## Author Contributions

ZW had full access to all of the data in the study. ZW and XZ took responsibility for the integrity of the data and the accuracy of data analyses. ZW and XZ designed the study. ZW, YY, JW, and YZ conducted the analyses and interpreted the data. ZW, YY, and JW drafted the manuscript. XZ had a guarantor. All authors revised the manuscript critically. All authors contributed to the article and approved the submitted version.

## Conflict of Interest

The authors declare that the research was conducted in the absence of any commercial or financial relationships that could be construed as a potential conflict of interest.

## Publisher’s Note

All claims expressed in this article are solely those of the authors and do not necessarily represent those of their affiliated organizations, or those of the publisher, the editors and the reviewers. Any product that may be evaluated in this article, or claim that may be made by its manufacturer, is not guaranteed or endorsed by the publisher.
