# Supplementary material for: Standardized Incidence Rate, Risk and Survival Outcomes of Second Primary Malignancy Among Renal Cell Carcinoma Survivors: A Nested Case-Control Study
Source: Front Oncol. 2021 Jul 30;11:716741. doi: 10.3389/fonc.2021.716741 (PMC8362854; doi:10.3389/fonc.2021.716741)
Supplement: Supplementary file 1 [file DataSheet_1.docx]

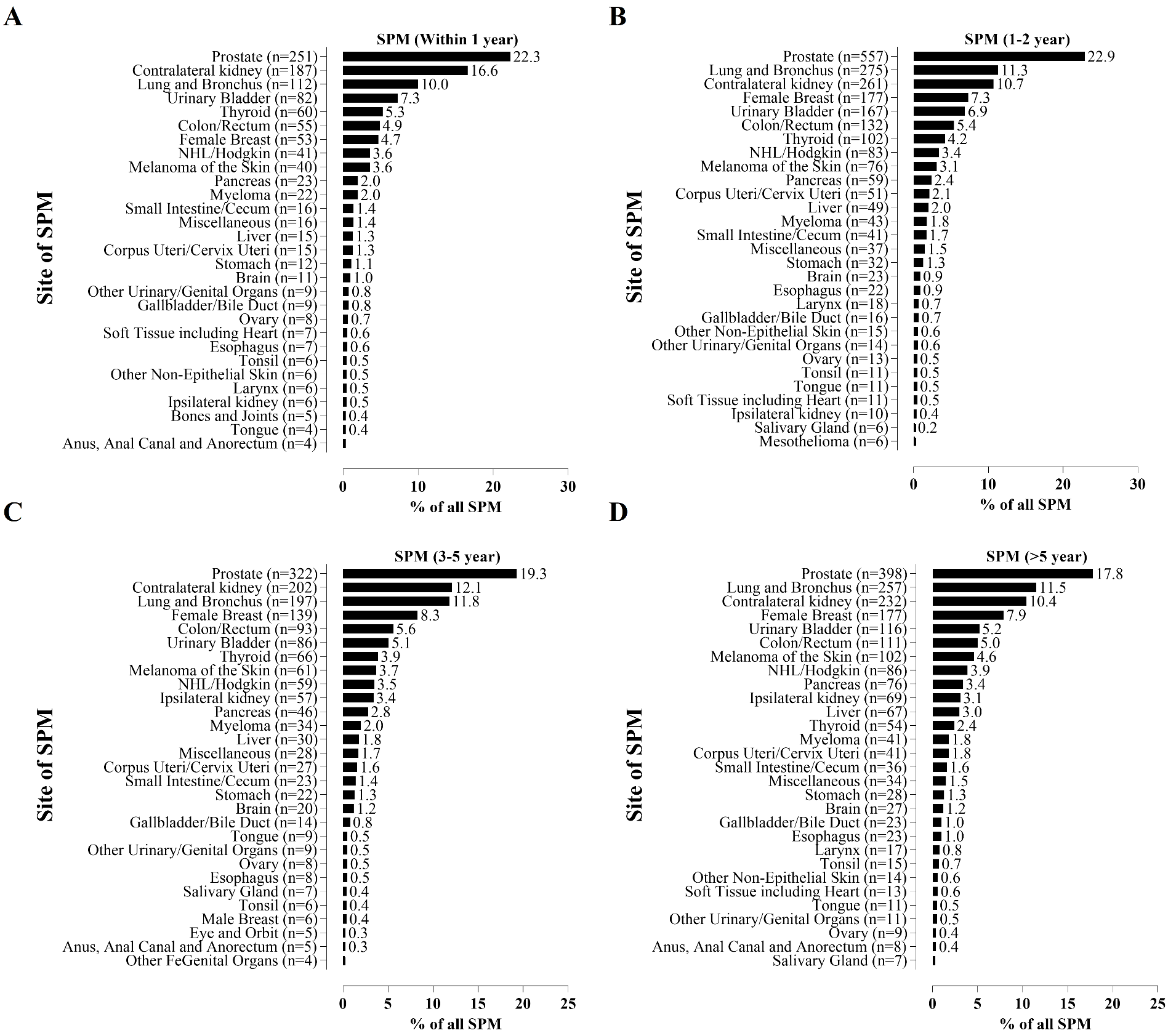


**Supplementary Figure 1.** Anatomic distribution of second primary malignancy (SPM) stratified by different time intervals after the diagnosis of the first primary renal cell carcinoma.

Note: For analyses of SPM distribution, we included crude data for 7461 patients with renal cell carcinoma by histologic confirmation, and just presented the top-29 sites of SPM. For analyses of the time interval to SPM, we included patients with the first primary renal cell carcinoma diagnosed between 2004 and 2007 for >10-year follow-up and sample size >10.


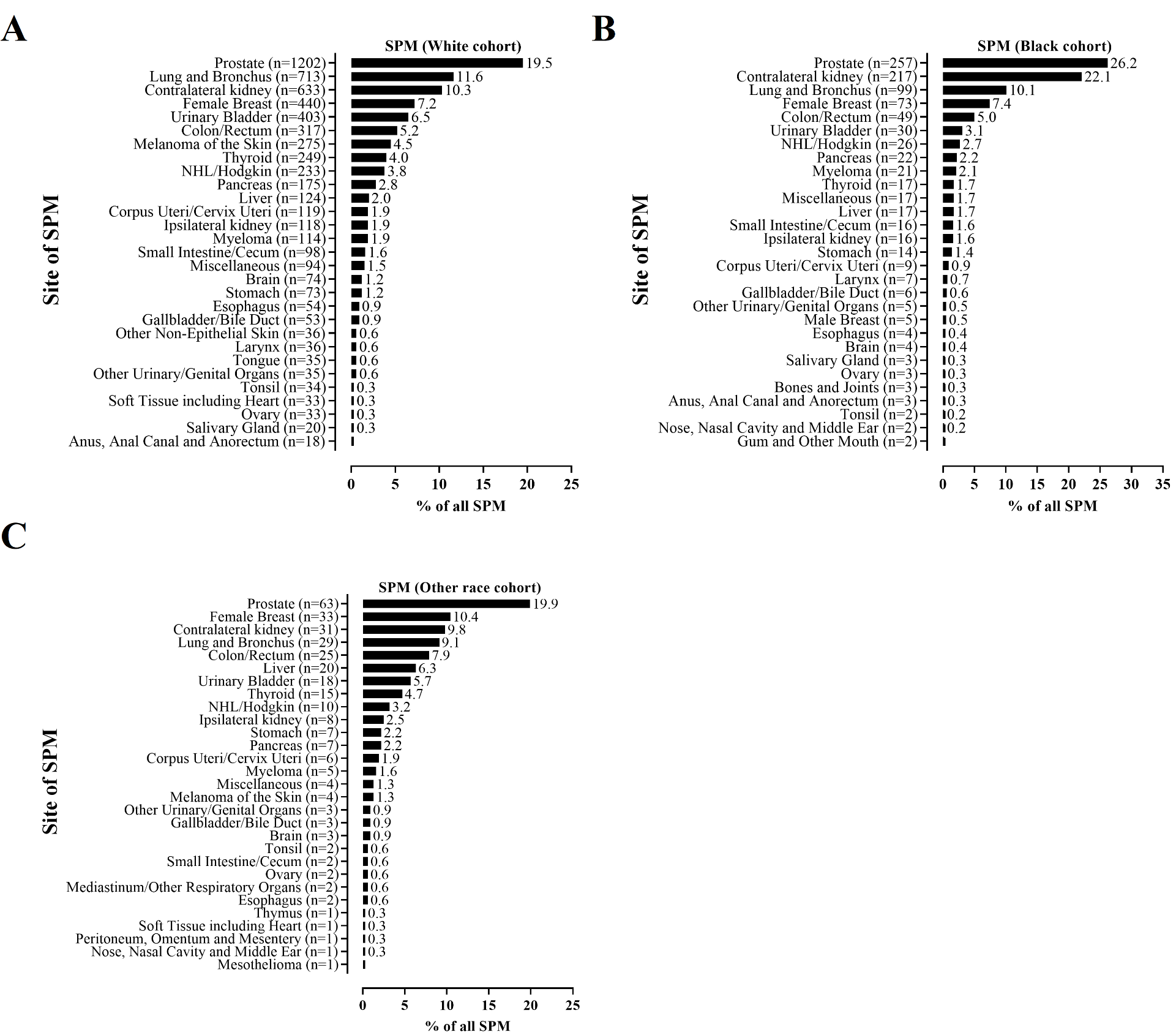
 **Supplementary Figure 2.** Anatomic distribution of second primary malignancy (SPM) stratified by the subgroup of ethnicity.

Note: For analyses of SPM distribution, we included crude data for 7,461 patients with renal cell carcinoma patients by histologic confirmation, and just presented the top-29 sites of SPM. For analyses of the time interval to SPM, we included patients with the first primary renal cell carcinoma diagnosed between 2004 and 2007 for >10-year follow-up and sample size >10.


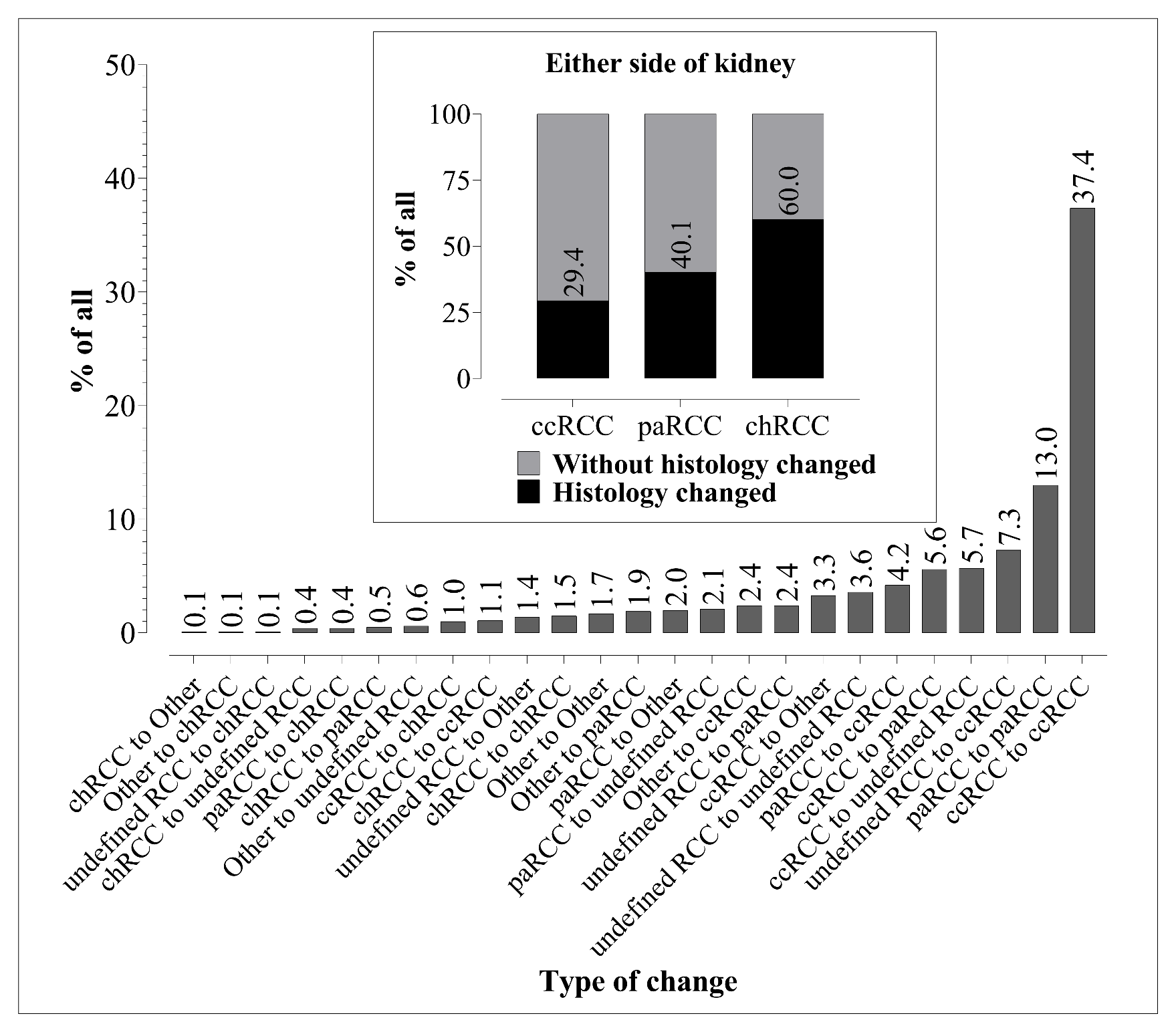


**Supplementary Figure 3.** Distribution of histologic changes between the first primary renal cell carcinoma and second primary renal cell carcinoma.

**Abbreviations:** ccRCC: clear-cell renal cell carcinoma; paRCC: papillary renal cell carcinoma; chRCC: chromophobe renal cell carcinoma.


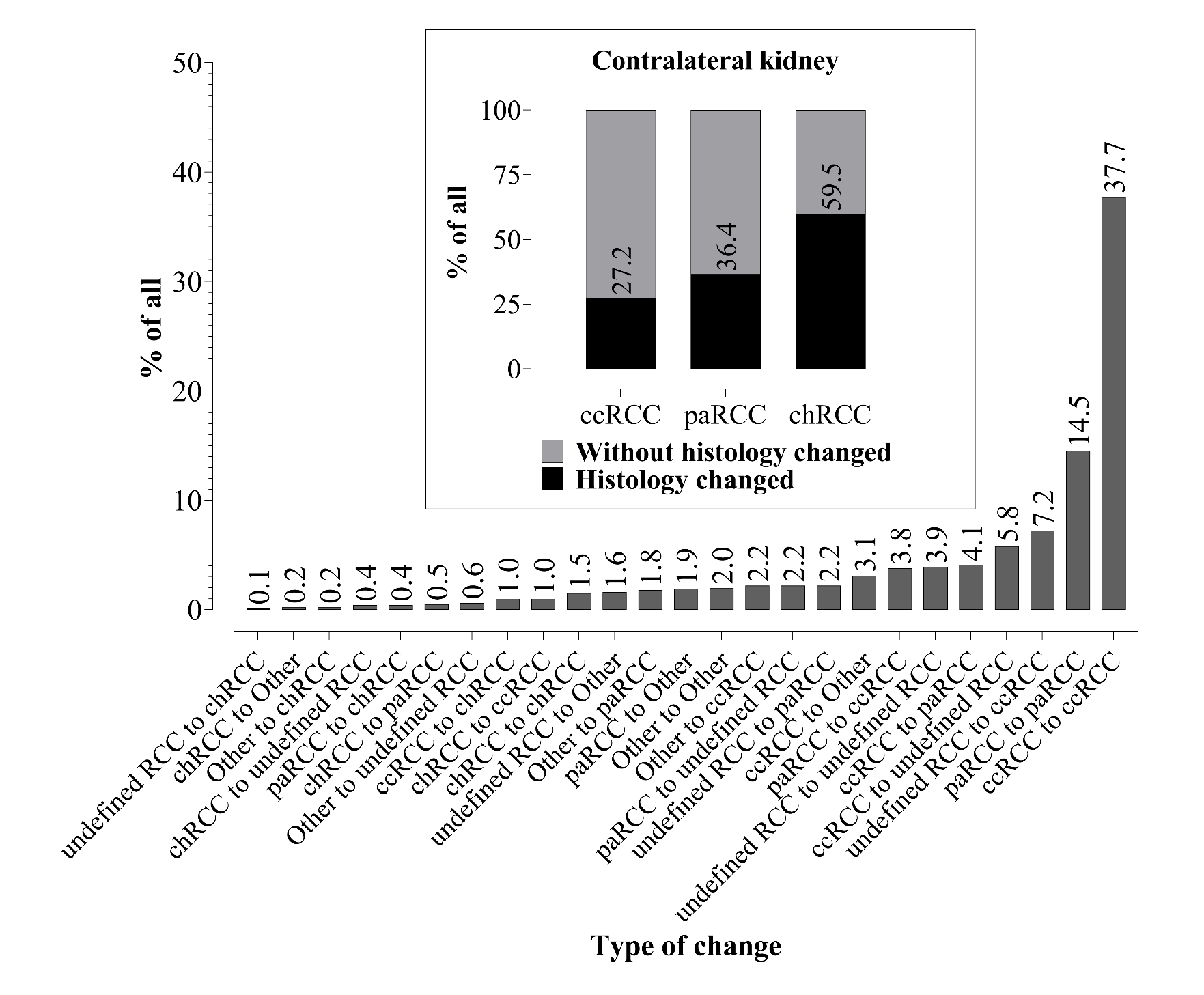


**Supplementary Figure 4.** Distribution of histologic changes between the first primary renal cell carcinoma and second primary contralateral renal cell carcinoma.

**Abbreviations:** ccRCC: clear-cell renal cell carcinoma; paRCC: papillary renal cell carcinoma; chRCC: chromophobe renal cell carcinoma.


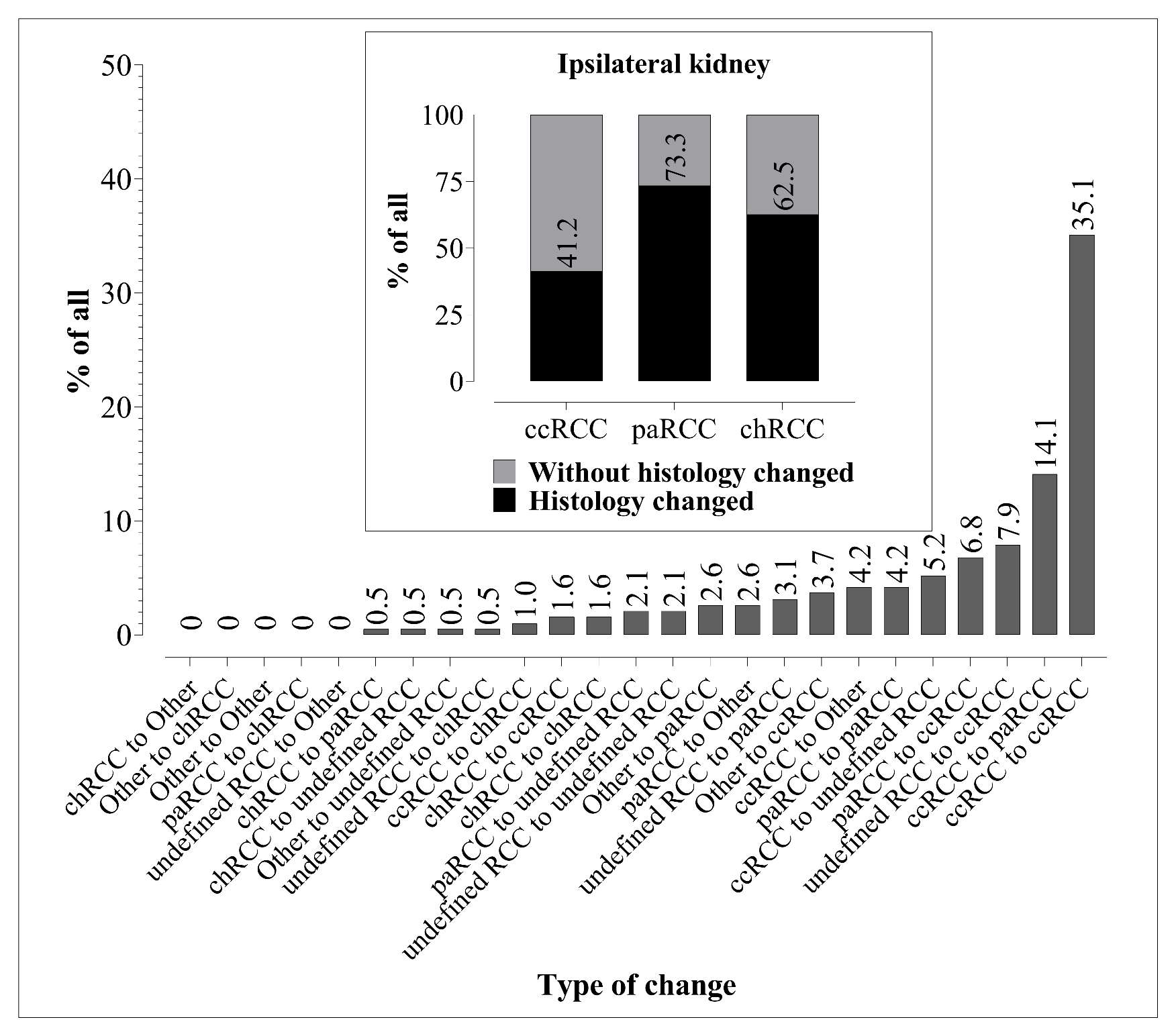


**Supplementary Figure 5.** Distribution of histologic changes between the first primary renal cell carcinoma and second primary ipsilateral renal cell carcinoma.

**Abbreviations:** ccRCC: clear-cell renal cell carcinoma; paRCC: papillary renal cell carcinoma; chRCC: chromophobe renal cell carcinoma.


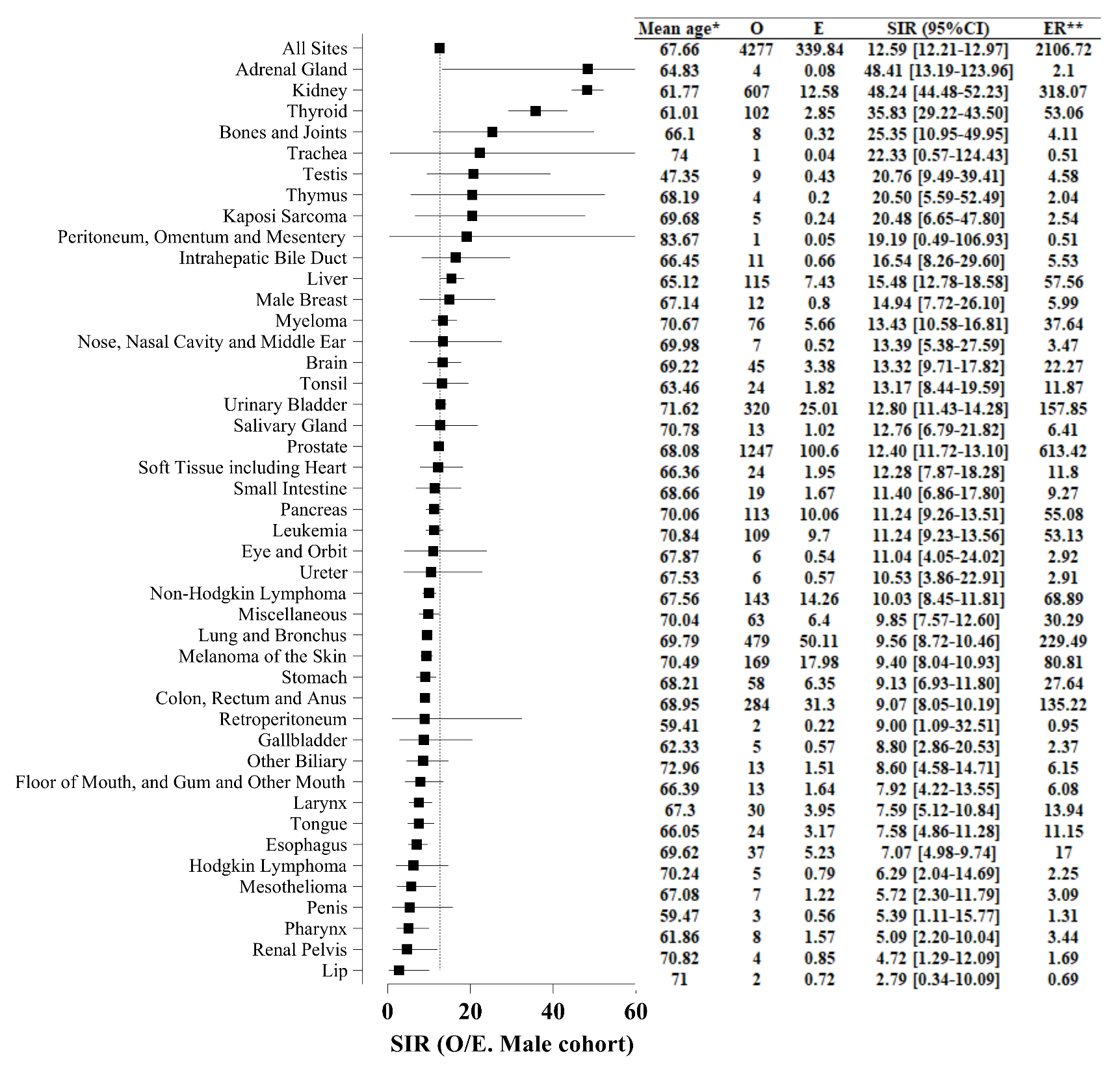


**Supplementary Figure 6.** Site site-specific standardized incidence ratio (SIR) of second primary malignancy (SPM) for a male cohort.

**Abbreviations**: O, observed; E, expected; ER: excess risk.

***Note:*** SIR was defined as the observed (O)-to-expected (E) ratio;

*Mean age at the event; **Excess risk is per 10,000.


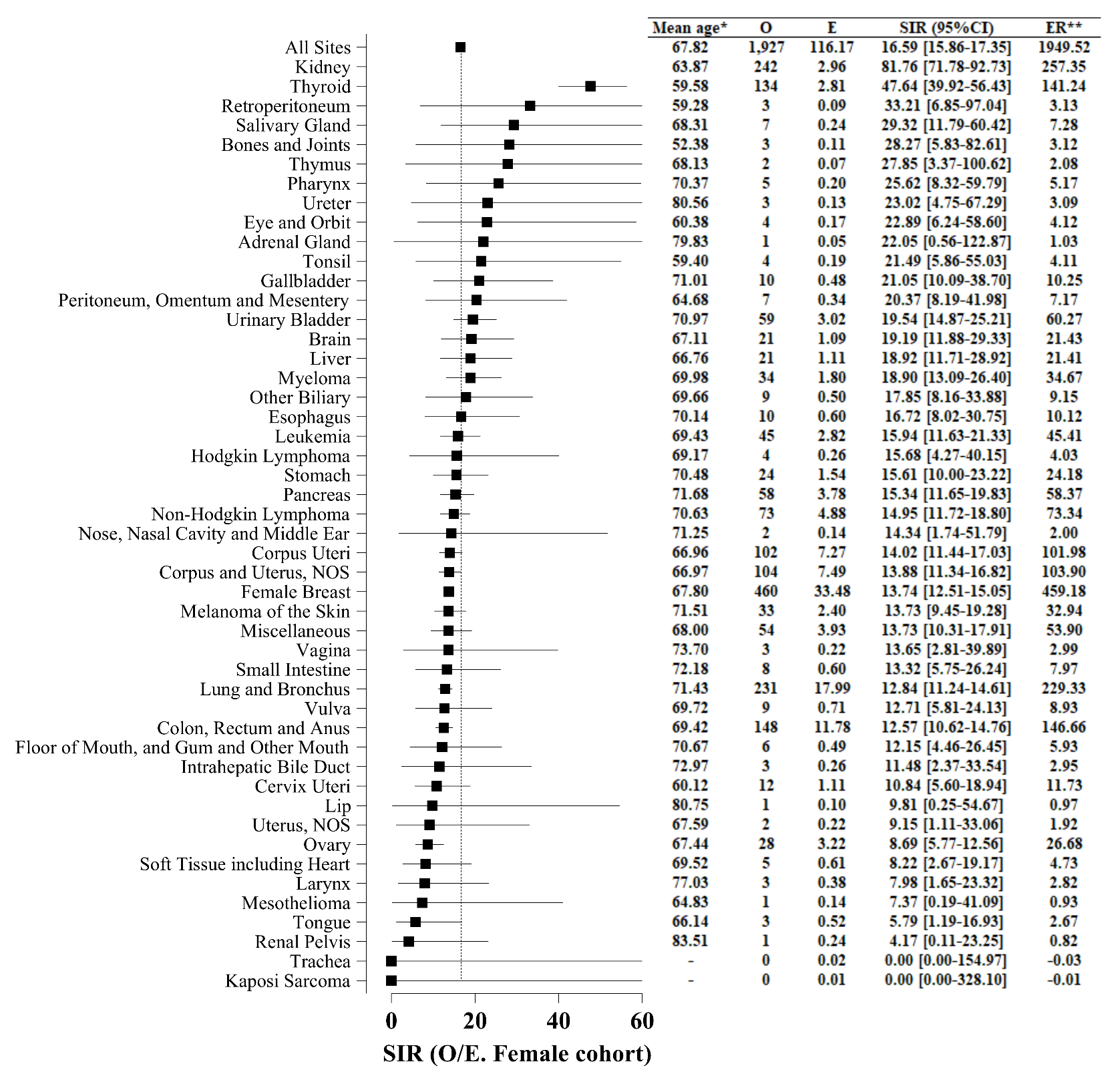


**Supplementary Figure 7.** Site-specific standardized incidence ratio (SIR) of second primary malignancy (SPM) for a female cohort.

**Abbreviations**: O, observed; E, expected; ER: excess risk.

***Note:*** SIR was defined as the observed (O)-to-expected (E) ratio;

*Mean age at the event; **Excess risk is per 10,000.
