# Supplementary material for: Standardized Incidence Rate, Risk and Survival Outcomes of Second Primary Malignancy Among Renal Cell Carcinoma Survivors: A Nested Case-Control Study
Source: Front Oncol. 2021 Jul 30;11:716741. doi: 10.3389/fonc.2021.716741 (PMC8362854; doi:10.3389/fonc.2021.716741)
Supplement: Supplementary file 2 [file Table_1.docx]

| Supplementary table 1. Demographic and clinic characteristics for patients with renal cell carcinoma as fist primary malignant tumor between 2004–2015 before propensity score matching. | | | | |  |
| --- | --- | --- | --- | --- | --- |
|  | **Overall** | **None-SPM (Control)** | **With SPM (Case)** | ***P*-value** |  |
|  | (N=62985) | (N=56781) | (N=6204) |  |  |
| **Year at diagnosis** | |  |  |  | <0.001 |
| 2004 | | 4087 (6.5%) | 3401 (6.0%) | 686 (11.1%) |  |
| 2005 | | 4216 (6.7%) | 3525 (6.2%) | 691 (11.1%) |  |
| 2006 | | 4715 (7.5%) | 3991 (7.0%) | 724 (11.7%) |  |
| 2007 | | 5145 (8.2%) | 4429 (7.8%) | 716 (11.5%) |  |
| 2008 | | 5546 (8.8%) | 4843 (8.5%) | 703 (11.3%) |  |
| 2009 | | 5713 (9.1%) | 5076 (8.9%) | 637 (10.3%) |  |
| 2010 | | 5328 (8.5%) | 4803 (8.5%) | 525 (8.5%) |  |
| 2011 | | 5360 (8.5%) | 4933 (8.7%) | 427 (6.9%) |  |
| 2012 | | 5515 (8.8%) | 5148 (9.1%) | 367 (5.9%) |  |
| 2013 | | 5464 (8.7%) | 5119 (9.0%) | 345 (5.6%) |  |
| 2014 | | 5816 (9.2%) | 5571 (9.8%) | 245 (3.9%) |  |
| 2015 | | 6080 (9.7%) | 5942 (10.5%) | 138 (2.2%) |  |
| **SEER register** | |  |  |  | <0.001 |
| Atlanta (Metropolitan) | | 1849 (2.9%) | 1644 (2.9%) | 205 (3.3%) |  |
| California excluding SF/SJM/LA | | 13634 (21.6%) | 12368 (21.8%) | 1266 (20.4%) |  |
| Connecticut | | 2589 (4.1%) | 2310 (4.1%) | 279 (4.5%) |  |
| Detroit (Metropolitan) | | 3490 (5.5%) | 3143 (5.5%) | 347 (5.6%) |  |
| Greater Georgia | | 4834 (7.7%) | 4357 (7.7%) | 477 (7.7%) |  |
| Hawaii | | 1124 (1.8%) | 1021 (1.8%) | 103 (1.7%) |  |
| Iowa | | 3077 (4.9%) | 2751 (4.8%) | 326 (5.3%) |  |
| Kentucky | | 4996 (7.9%) | 4488 (7.9%) | 508 (8.2%) |  |
| Los Angeles | | 5900 (9.4%) | 5363 (9.4%) | 537 (8.7%) |  |
| Louisiana | | 4459 (7.1%) | 4017 (7.1%) | 442 (7.1%) |  |
| New Jersey | | 6047 (9.6%) | 5310 (9.4%) | 737 (11.9%) |  |
| New Mexico | | 1336 (2.1%) | 1259 (2.2%) | 77 (1.2%) |  |
| Rural Georgia | | 122 (0.2%) | 110 (0.2%) | 12 (0.2%) |  |
| San Francisco Oakland SMSA | | 2759 (4.4%) | 2499 (4.4%) | 260 (4.2%) |  |
| San Jose Monterey | | 1498 (2.4%) | 1354 (2.4%) | 144 (2.3%) |  |
| Seattle (Puget Sound) | | 3711 (5.9%) | 3339 (5.9%) | 372 (6.0%) |  |
| Utah | | 1560 (2.5%) | 1448 (2.6%) | 112 (1.8%) |  |
| **Marital status** | |  |  |  | <0.001 |
| Married | | 41326 (65.6%) | 37055 (65.3%) | 4271 (68.8%) |  |
| Single/unmarried | | 9897 (15.7%) | 9124 (16.1%) | 773 (12.5%) |  |
| Widowed/Divorced/Separated | | 11762 (18.7%) | 10602 (18.7%) | 1160 (18.7%) |  |
| **Population density** | |  |  |  | 0.394 |
| Counties | | 55027 (87.4%) | 49584 (87.3%) | 5443 (87.7%) |  |
| Rural | | 992 (1.6%) | 906 (1.6%) | 86 (1.4%) |  |
| Urban | | 6966 (11.1%) | 6291 (11.1%) | 675 (10.9%) |  |
| **Region** | |  |  |  | <0.001 |
| East | | 24896 (39.5%) | 22236 (39.2%) | 2660 (42.9%) |  |
| Northern Plains | | 6567 (10.4%) | 5894 (10.4%) | 673 (10.8%) |  |
| Pacific Coast | | 28626 (45.4%) | 25944 (45.7%) | 2682 (43.2%) |  |
| Southwest | | 2896 (4.6%) | 2707 (4.8%) | 189 (3.0%) |  |
| **Median family income quartile. $** | |  |  |  | <0.001 |
| 1 or less | | 14129 (22.4%) | 12853 (22.6%) | 1276 (20.6%) |  |
| 1 ~ 2 | | 17027 (27.0%) | 15409 (27.1%) | 1618 (26.1%) |  |
| 2 ~ 3 | | 16210 (25.7%) | 14507 (25.5%) | 1703 (27.5%) |  |
| 3 ~ 4 | | 15619 (24.8%) | 14012 (24.7%) | 1607 (25.9%) |  |
| **Age at diagnosis, years** | |  |  |  | <0.001 |
| Mean (SD, [Min-Max]) | | 59.8 (12.5, [18.0-100]) | 59.4 (12.6, [18.0-100]) | 63.3 (10.7, [26.0-93.0]) |  |
| Median (Q1, Q3) | | 60.0 (51.0, 69.0) | 60.0 (51.0, 68.0) | 64.0 (56.0, 71.0) |  |
| **Age at diagnosis, years** | |  |  |  | <0.001 |
| (~44] | | 7461 (11.8%) | 7176 (12.6%) | 285 (4.6%) |  |
| (45-59] | | 22737 (36.1%) | 20840 (36.7%) | 1897 (30.6%) |  |
| (60-74] | | 24968 (39.6%) | 21911 (38.6%) | 3057 (49.3%) |  |
| (75~) | | 7819 (12.4%) | 6854 (12.1%) | 965 (15.6%) |  |
| **Race** | |  |  |  | <0.001 |
| White | | 52233 (82.9%) | 47087 (82.9%) | 5146 (82.9%) |  |
| Black | | 6882 (10.9%) | 6108 (10.8%) | 774 (12.5%) |  |
| Other | | 3870 (6.1%) | 3586 (6.3%) | 284 (4.6%) |  |
| **Sex** | |  |  |  | <0.001 |
| Female | | 23702 (37.6%) | 21775 (38.3%) | 1927 (31.1%) |  |
| Male | | 39283 (62.4%) | 35006 (61.7%) | 4277 (68.9%) |  |
| **Grade** | |  |  |  | <0.001 |
| Grade I | | 8375 (13.3%) | 7513 (13.2%) | 862 (13.9%) |  |
| Grade II | | 33939 (53.9%) | 30485 (53.7%) | 3454 (55.7%) |  |
| Grade III/IV | | 20671 (32.8%) | 18783 (33.1%) | 1888 (30.4%) |  |
| **Tumor side** | |  |  |  | 0.579 |
| Left | | 30810 (48.9%) | 27754 (48.9%) | 3056 (49.3%) |  |
| Right | | 32175 (51.1%) | 29027 (51.1%) | 3148 (50.7%) |  |
| **Histological type** | |  |  |  | <0.001 |
| ccRCC | | 40072 (63.6%) | 36396 (64.1%) | 3676 (59.3%) |  |
| chRCC | | 2966 (4.7%) | 2721 (4.8%) | 245 (3.9%) |  |
| paRCC | | 7283 (11.6%) | 6403 (11.3%) | 880 (14.2%) |  |
| RCC (undefined) | | 9408 (14.9%) | 8383 (14.8%) | 1025 (16.5%) |  |
| Other type RCC | | 3256 (5.2%) | 2878 (5.1%) | 378 (6.1%) |  |
| **AJCC stage group** | |  |  |  | <0.001 |
| I | | 44694 (71.0%) | 40158 (70.7%) | 4536 (73.1%) |  |
| II | | 6821 (10.8%) | 6164 (10.9%) | 657 (10.6%) |  |
| III/IV | | 11470 (18.2%) | 10459 (18.4%) | 1011 (16.3%) |  |
| **AJCC T stage** | |  |  |  | <0.001 |
| T1 | | 44847 (71.2%) | 40300 (71.0%) | 4547 (73.3%) |  |
| T2 | | 6980 (11.1%) | 6315 (11.1%) | 665 (10.7%) |  |
| T3/T4 | | 11158 (17.7%) | 10166 (17.9%) | 992 (16.0%) |  |
| **AJCC N stage** | |  |  |  | <0.001 |
| N0 | | 61940 (98.3%) | 55788 (98.3%) | 6152 (99.2%) |  |
| N1 | | 1045 (1.7%) | 993 (1.7%) | 52 (0.8%) |  |
| **Surgery** | |  |  |  | <0.001 |
| Cryosurgery/Radiofrequency ablation | | 1541 (2.4%) | 1341 (2.4%) | 200 (3.2%) |  |
| Nephrectomy | | 846 (1.3%) | 747 (1.3%) | 99 (1.6%) |  |
| Partial nephrectomy | | 20656 (32.8%) | 18779 (33.1%) | 1877 (30.3%) |  |
| Radical nephrectomy | | 39942 (63.4%) | 35914 (63.3%) | 4028 (64.9%) |  |
| **Tumor size, mm** | |  |  |  |  |
| Mean (SD, [Min-Max]) | | 49.0 (28.9, [1.00-150]) | 49.2 (29.1, [1.00-150]) | 47.5 (27.1, [1.00-150]) |  |
| Median (Q1, Q3) | | 41.0 (27.0, 65.0) | 41.0 (27.0, 65.0) | 40.0 (28.0, 60.0) |  |
| **Tumor size** | |  |  |  | <0.001 |
| (~4] cm | | 31234 (49.6%) | 28079 (49.5%) | 3155 (50.9%) |  |
| (4.1-7] cm | | 19282 (30.6%) | 17298 (30.5%) | 1984 (32.0%) |  |
| (7.1-10] cm | | 8453 (13.4%) | 7704 (13.6%) | 749 (12.1%) |  |
| (10~) cm | | 4016 (6.4%) | 3700 (6.5%) | 316 (5.1%) |  |
| **SPM onset** | |  |  |  | / |
| None-SPM | | 56781 (90.2%) | 56781 (100%) | / |  |
| With contralateral RCC SPM | | 747 (1.2%) | / | 747 (12.0%) |  |
| With ipsilateral RCC SPM | | 101 (0.2%) | / | 101 (1.6%) |  |
| With other SPM | | 3703 (5.9%) | / | 3703 (59.7%) |  |
| With other urological system SPM | | 1653 (2.6%) | / | 1653 (26.6%) |  |
| SPM, second primary malignancy; AJCC, American Joint Committee on Cancer System; ccRCC, clear cell renal cell carcinoma; paRCC, papillary renal cell carcinoma; chRCC, chromophobe renal cell carcinoma. *. Matched by year of diagnosis and SEER register by propensity score matching at 1:5. | | | | |  |

| Supplementary Table 2. Demographic and clinic characteristics for patients with renal cell carcinoma as the fist primary malignant tumor between 2004–2015 after propensity score matching*. | | | | | | | | | | | | |
| --- | --- | --- | --- | --- | --- | --- | --- | --- | --- | --- | --- | --- |
|  | **Case-control group 1** | | |  | **Case-control group 2** | | |  | **Case-control group 3** | | | |
|  | **None-SPM** | **Non-RCC SPM** | ***p-value*** |  | **None-SPM** | **SPM of contralateral RCC** | ***p-value*** |  | **None-SPM** | **SPM of ipsilateral RCC**** | ***p-value*** |  |
|  | (N=26780) | (N=5356) |  |  | (N=3735) | (N=747) |  |  | (N=410) | (N=82) |  |  |
| **Year at diagnosis** |  |  | 1.000 |  |  |  | 1.000 |  |  |  | 1.000 |  |
| 2004 | 3066 (11.4%) | 615 (11.5%) |  |  | 300 (8.0%) | 60 (8.0%) |  |  | 40 (9.8%) | 8 (9.8%) |  |  |
| 2005 | 3033 (11.3%) | 606 (11.3%) |  |  | 380 (10.2%) | 76 (10.2%) |  |  | 35 (8.5%) | 7 (8.5%) |  |  |
| 2006 | 3133 (11.7%) | 621 (11.6%) |  |  | 460 (12.3%) | 92 (12.3%) |  |  | 55 (13.4%) | 11 (13.4%) |  |  |
| 2007 | 3038 (11.3%) | 614 (11.5%) |  |  | 455 (12.2%) | 91 (12.2%) |  |  | 50 (12.2%) | 10 (12.2%) |  |  |
| 2008 | 3050 (11.4%) | 608 (11.4%) |  |  | 410 (11.0%) | 82 (11.0%) |  |  | 55 (13.4%) | 11 (13.4%) |  |  |
| 2009 | 2755 (10.3%) | 551 (10.3%) |  |  | 360 (9.6%) | 72 (9.6%) |  |  | 70 (17.1%) | 14 (17.1%) |  |  |
| 2010 | 2305 (8.6%) | 461 (8.6%) |  |  | 270 (7.2%) | 54 (7.2%) |  |  | 50 (12.2%) | 10 (12.2%) |  |  |
| 2011 | 1815 (6.8%) | 363 (6.8%) |  |  | 270 (7.2%) | 54 (7.2%) |  |  | 25 (6.1%) | 5 (6.1%) |  |  |
| 2012 | 1530 (5.7%) | 306 (5.7%) |  |  | 265 (7.1%) | 53 (7.1%) |  |  | 15 (3.7%) | 3 (3.7%) |  |  |
| 2013 | 1440 (5.4%) | 288 (5.4%) |  |  | 275 (7.4%) | 55 (7.4%) |  |  | 10 (2.4%) | 2 (2.4%) |  |  |
| 2014 | 1000 (3.7%) | 200 (3.7%) |  |  | 220 (5.9%) | 44 (5.9%) |  |  | 0 (0%) | 0 (0%) |  |  |
| 2015 | 615 (2.3%) | 123 (2.3%) |  |  | 70 (1.9%) | 14 (1.9%) |  |  | 5 (1.2%) | 1 (1.2%) |  |  |
| **SEER register** |  |  | 1.000 |  |  |  | 1.000 |  |  |  | 1.000 |  |
| Atlanta (Metropolitan) | 832 (3.1%) | 166 (3.1%) |  |  | 185 (5.0%) | 37 (5.0%) |  |  | 5 (1.2%) | 1 (1.2%) |  |  |
| California excluding SF/SJM/LA | 5555 (20.7%) | 1109 (20.7%) |  |  | 710 (19.0%) | 142 (19.0%) |  |  | 75 (18.3%) | 15 (18.3%) |  |  |
| Connecticut | 1208 (4.5%) | 245 (4.6%) |  |  | 155 (4.1%) | 31 (4.1%) |  |  | 15 (3.7%) | 3 (3.7%) |  |  |
| Detroit (Metropolitan) | 1410 (5.3%) | 282 (5.3%) |  |  | 285 (7.6%) | 57 (7.6%) |  |  | 30 (7.3%) | 6 (7.3%) |  |  |
| Greater Georgia | 2060 (7.7%) | 407 (7.6%) |  |  | 325 (8.7%) | 65 (8.7%) |  |  | 25 (6.1%) | 5 (6.1%) |  |  |
| Hawaii | 438 (1.6%) | 96 (1.8%) |  |  | 30 (0.8%) | 6 (0.8%) |  |  | 5 (1.2%) | 1 (1.2%) |  |  |
| Iowa | 1364 (5.1%) | 280 (5.2%) |  |  | 200 (5.4%) | 40 (5.4%) |  |  | 15 (3.7%) | 3 (3.7%) |  |  |
| Kentucky | 2243 (8.4%) | 442 (8.3%) |  |  | 265 (7.1%) | 53 (7.1%) |  |  | 55 (13.4%) | 11 (13.4%) |  |  |
| Los Angeles | 2297 (8.6%) | 453 (8.5%) |  |  | 360 (9.6%) | 72 (9.6%) |  |  | 40 (9.8%) | 8 (9.8%) |  |  |
| Louisiana | 1937 (7.2%) | 387 (7.2%) |  |  | 250 (6.7%) | 50 (6.7%) |  |  | 15 (3.7%) | 3 (3.7%) |  |  |
| New Jersey | 3136 (11.7%) | 628 (11.7%) |  |  | 465 (12.4%) | 93 (12.4%) |  |  | 75 (18.3%) | 15 (18.3%) |  |  |
| New Mexico | 345 (1.3%) | 69 (1.3%) |  |  | 35 (0.9%) | 7 (0.9%) |  |  | 5 (1.2%) | 1 (1.2%) |  |  |
| Rural Georgia | 55 (0.2%) | 10 (0.2%) |  |  | 10 (0.3%) | 2 (0.3%) |  |  | 0 (0%) | 0 (0%) |  |  |
| San Francisco Oakland SMSA | 1132 (4.2%) | 224 (4.2%) |  |  | 165 (4.4%) | 33 (4.4%) |  |  | 15 (3.7%) | 3 (3.7%) |  |  |
| San Jose Monterey | 616 (2.3%) | 129 (2.4%) |  |  | 75 (2.0%) | 15 (2.0%) |  |  | 0 (0%) | 0 (0%) |  |  |
| Seattle (Puget Sound) | 1662 (6.2%) | 329 (6.1%) |  |  | 170 (4.6%) | 34 (4.6%) |  |  | 30 (7.3%) | 6 (7.3%) |  |  |
| Utah | 490 (1.8%) | 100 (1.9%) |  |  | 50 (1.3%) | 10 (1.3%) |  |  | 5 (1.2%) | 1 (1.2%) |  |  |
| **Marital status** |  |  | <0.001 |  |  |  | 0.002 |  |  |  | 0.235 |  |
| Married | 17681 (66.0%) | 3668 (68.5%) |  |  | 2463 (65.9%) | 523 (70.0%) |  |  | 277 (67.6%) | 63 (76.8%) |  |  |
| Single/unmarried | 4044 (15.1%) | 643 (12.0%) |  |  | 550 (14.7%) | 121 (16.2%) |  |  | 63 (15.4%) | 8 (9.8%) |  |  |
| Widowed/Divorced/Separated | 5055 (18.9%) | 1045 (19.5%) |  |  | 722 (19.3%) | 103 (13.8%) |  |  | 70 (17.1%) | 11 (13.4%) |  |  |
| **Population** |  |  | 0.495 |  |  |  | 0.465 |  |  |  | 0.576 |  |
| Counties | 23364 (87.2%) | 4691 (87.6%) |  |  | 3271 (87.6%) | 665 (89.0%) |  |  | 370 (90.2%) | 71 (86.6%) |  |  |
| Rural | 432 (1.6%) | 75 (1.4%) |  |  | 68 (1.8%) | 10 (1.3%) |  |  | 5 (1.2%) | 1 (1.2%) |  |  |
| Urban | 2984 (11.1%) | 590 (11.0%) |  |  | 396 (10.6%) | 72 (9.6%) |  |  | 35 (8.5%) | 10 (12.2%) |  |  |
| **Region** |  |  | 0.988 |  |  |  | 1.000 |  |  |  | 1.000 |  |
| East | 11471 (42.8%) | 2285 (42.7%) |  |  | 1655 (44.3%) | 331 (44.3%) |  |  | 190 (46.3%) | 38 (46.3%) |  |  |
| Northern Plains | 2774 (10.4%) | 562 (10.5%) |  |  | 485 (13.0%) | 97 (13.0%) |  |  | 45 (11.0%) | 9 (11.0%) |  |  |
| Pacific Coast | 11700 (43.7%) | 2340 (43.7%) |  |  | 1510 (40.4%) | 302 (40.4%) |  |  | 165 (40.2%) | 33 (40.2%) |  |  |
| Southwest | 835 (3.1%) | 169 (3.2%) |  |  | 85 (2.3%) | 17 (2.3%) |  |  | 10 (2.4%) | 2 (2.4%) |  |  |
| **Median family income quartile. $** |  |  | 0.061 |  |  |  | 0.602 |  |  |  | 0.452 |  |
| 1 or less | 5889 (22.0%) | 1099 (20.5%) |  |  | 833 (22.3%) | 157 (21.0%) |  |  | 98 (23.9%) | 15 (18.3%) |  |  |
| 1 ~ 2 | 7086 (26.5%) | 1409 (26.3%) |  |  | 953 (25.5%) | 181 (24.2%) |  |  | 103 (25.1%) | 26 (31.7%) |  |  |
| 2 ~ 3 | 7009 (26.2%) | 1469 (27.4%) |  |  | 958 (25.6%) | 206 (27.6%) |  |  | 109 (26.6%) | 24 (29.3%) |  |  |
| 3 ~ 4 | 6796 (25.4%) | 1379 (25.7%) |  |  | 991 (26.5%) | 203 (27.2%) |  |  | 100 (24.4%) | 17 (20.7%) |  |  |
| **Age at diagnosis, years** |  |  | <0.001 |  |  |  | 0.001 |  |  |  | 0.120 |  |
| Mean (SD [Min-Max]) | 59.4 (12.7[18.0-97.0]) | 64.2 (10.3[27.0-93.0]) |  |  | 59.5 (12.6[21.0-93.0]) | 57.9 (11.2[26.0-86.0]) |  |  | 57.3 (12.5[20.0-87.0]) | 59.7 (11.4[34.0-84.0]) |  |  |
| Median (Q1, Q3) | 60.0 (51.0, 68.0) | 65.0 (57.0, 72.0) |  |  | 60.0 (51.0, 69.0) | 58.0 (50.0, 66.0) |  |  | 58.0 (49.0, 66.0) | 59.0 (51.3, 69.0) |  |  |
| **Age at diagnosis, years** |  |  | <0.001 |  |  |  | 0.001 |  |  |  | 0.179 |  |
| (~44] | 3437 (12.8%) | 184 (3.4%) |  |  | 461 (12.3%) | 93 (12.4%) |  |  | 67 (16.3%) | 6 (7.3%) |  |  |
| (45-59] | 9925 (37.1%) | 1540 (28.8%) |  |  | 1374 (36.8%) | 313 (41.9%) |  |  | 161 (39.3%) | 37 (45.1%) |  |  |
| (60-74] | 10026 (37.4%) | 2734 (51.0%) |  |  | 1448 (38.8%) | 285 (38.2%) |  |  | 148 (36.1%) | 30 (36.6%) |  |  |
| (75~) | 3392 (12.7%) | 898 (16.8%) |  |  | 452 (12.1%) | 56 (7.5%) |  |  | 34 (8.3%) | 9 (11.0%) |  |  |
| **Race** |  |  | 0.004 |  |  |  | <0.001 |  |  |  | 0.893 |  |
| White | 22502 (84.0%) | 4513 (84.3%) |  |  | 3118 (83.5%) | 546 (73.1%) |  |  | 358 (87.3%) | 71 (86.6%) |  |  |
| Black | 2766 (10.3%) | 595 (11.1%) |  |  | 413 (11.1%) | 172 (23.0%) |  |  | 32 (7.8%) | 6 (7.3%) |  |  |
| Other | 1512 (5.6%) | 248 (4.6%) |  |  | 204 (5.5%) | 29 (3.9%) |  |  | 20 (4.9%) | 5 (6.1%) |  |  |
| **Sex** |  |  | <0.001 |  |  |  | <0.001 |  |  |  | 0.167 |  |
| Female | 10410 (38.9%) | 1686 (31.5%) |  |  | 1422 (38.1%) | 211 (28.2%) |  |  | 156 (38.0%) | 24 (29.3%) |  |  |
| Male | 16370 (61.1%) | 3670 (68.5%) |  |  | 2313 (61.9%) | 536 (71.8%) |  |  | 254 (62.0%) | 58 (70.7%) |  |  |
| **Grade** |  |  | 0.012 |  |  |  | 0.151 |  |  |  | 0.244 |  |
| Grade I | 3810 (14.2%) | 749 (14.0%) |  |  | 537 (14.4%) | 96 (12.9%) |  |  | 94 (22.9%) | 13 (15.9%) |  |  |
| Grade II | 14399 (53.8%) | 2993 (55.9%) |  |  | 2027 (54.3%) | 391 (52.3%) |  |  | 251 (61.2%) | 58 (70.7%) |  |  |
| Grade III/IV | 8571 (32.0%) | 1614 (30.1%) |  |  | 1171 (31.4%) | 260 (34.8%) |  |  | 65 (15.9%) | 11 (13.4%) |  |  |
| **Tumor side** |  |  | 1 |  |  |  | 0.329 |  |  |  | 0.331 |  |
| Left | 13142 (49.1%) | 2629 (49.1%) |  |  | 1804 (48.3%) | 376 (50.3%) |  |  | 183 (44.6%) | 42 (51.2%) |  |  |
| Right | 13638 (50.9%) | 2727 (50.9%) |  |  | 1931 (51.7%) | 371 (49.7%) |  |  | 227 (55.4%) | 40 (48.8%) |  |  |
| **Histological type** |  |  | <0.001 |  |  |  | <0.001 |  |  |  | 0.259 |  |
| ccRCC | 16370 (61.1%) | 3185 (59.5%) |  |  | 2267 (60.7%) | 421 (56.4%) |  |  | 236 (57.6%) | 56 (68.3%) |  |  |
| chRCC | 1310 (4.9%) | 216 (4.0%) |  |  | 201 (5.4%) | 26 (3.5%) |  |  | 14 (3.4%) | 2 (2.4%) |  |  |
| paRCC | 2910 (10.9%) | 718 (13.4%) |  |  | 427 (11.4%) | 150 (20.1%) |  |  | 64 (15.6%) | 9 (11.0%) |  |  |
| RCC (undefined) | 4743 (17.7%) | 904 (16.9%) |  |  | 629 (16.8%) | 110 (14.7%) |  |  | 80 (19.5%) | 10 (12.2%) |  |  |
| Other type RCC | 1447 (5.4%) | 333 (6.2%) |  |  | 211 (5.6%) | 40 (5.4%) |  |  | 16 (3.9%) | 5 (6.1%) |  |  |
| **AJCC stage group** |  |  | <0.001 |  |  |  | 0.263 |  |  |  | / |  |
| I | 18806 (70.2%) | 3902 (72.9%) |  |  | 2604 (69.7%) | 541 (72.4%) |  |  | 410 (100%) | 82 (100%) |  |  |
| II | 3022 (11.3%) | 574 (10.7%) |  |  | 420 (11.2%) | 82 (11.0%) |  |  |  |  |  |  |
| III/IV | 4952 (18.5%) | 880 (16.4%) |  |  | 711 (19.0%) | 124 (16.6%) |  |  |  |  |  |  |
| **AJCC T stage** |  |  | 0.001 |  |  |  | 0.327 |  |  |  | / |  |
| T1 | 18882 (70.5%) | 3911 (73.0%) |  |  | 2614 (70.0%) | 542 (72.6%) |  |  | 410 (100%) | 82 (100%) |  |  |
| T2 | 3105 (11.6%) | 581 (10.8%) |  |  | 432 (11.6%) | 83 (11.1%) |  |  |  |  |  |  |
| T3/T4 | 4793 (17.9%) | 864 (16.1%) |  |  | 689 (18.4%) | 122 (16.3%) |  |  |  |  |  |  |
| **AJCC N stage** |  |  | <0.001 |  |  |  | 0.011 |  |  |  | / |  |
| N0 | 26273 (98.1%) | 5310 (99.1%) |  |  | 3655 (97.9%) | 742 (99.3%) |  |  | 410 (100%) | 82 (100%) |  |  |
| N1 | 507 (1.9%) | 46 (0.9%) |  |  | 80 (2.1%) | 5 (0.7%) |  |  |  |  |  |  |
| **Surgery** |  |  | <0.001 |  |  |  | 0.005 |  |  |  | <0.001 |  |
| Cryosurgery/Radiofrequency ablation | 525 (2.0%) | 167 (3.1%) |  |  | 92 (2.5%) | 14 (1.9%) |  |  | 33 (8.0%) | 18 (22.0%) |  |  |
| Nephrectomy | 400 (1.5%) | 93 (1.7%) |  |  | 58 (1.6%) | 6 (0.8%) |  |  | 377 (92.0%) | 64 (78.0%) |  |  |
| Partial nephrectomy | 7698 (28.7%) | 1532 (28.6%) |  |  | 1090 (29.2%) | 263 (35.2%) |  |  |  |  |  |  |
| Radical nephrectomy | 18157 (67.8%) | 3564 (66.5%) |  |  | 2495 (66.8%) | 464 (62.1%) |  |  |  |  |  |  |
| **Tumor size, mm** |  |  | <0.001 |  |  |  | 0.685 |  |  |  | 0.330 |  |
| Mean (SD [Min-Max]) | 49.6 (29.1[1.00-150]) | 47.5 (26.9[1.00-150]) |  |  | 49.4 (28.7[2.00-150]) | 49.8 (29.5[3.00-150]) |  |  | 24.6 (8.34[3.00-40.0]) | 25.6 (7.99[5.00-40.0]) |  |  |
| Median (Q1, Q3) | 42.0 (28.0, 65.0) | 40.0 (28.0, 60.0) |  |  | 42.0 (28.0, 65.0) | 42.0 (28.0, 68.0) |  |  | 25.0 (18.0, 30.0) | 25.0 (20.3, 30.0) |  |  |
| **Tumor size** |  |  | <0.001 |  |  |  | 0.864 |  |  |  | / |  |
| (~4] cm | 13100 (48.9%) | 2710 (50.6%) |  |  | 1823 (48.8%) | 360 (48.2%) |  |  | 410 (100%) | 82 (100%) |  |  |
| (4.1-7] cm | 8204 (30.6%) | 1738 (32.4%) |  |  | 1166 (31.2%) | 231 (30.9%) |  |  |  |  |  |  |
| (7.1-10] cm | 3752 (14.0%) | 642 (12.0%) |  |  | 526 (14.1%) | 106 (14.2%) |  |  |  |  |  |  |
| (10~) cm | 1724 (6.4%) | 266 (5.0%) |  |  | 220 (5.9%) | 50 (6.7%) |  |  |  |  |  |  |
| RCC, renal cell carcinoma; SPM, second primary malignancy; AJCC, American Joint Committee on Cancer System;  *. Matched by year of diagnosis and SEER register by propensity score matching at 1:5. **First primary renal cell carcinoma with AJCC stage of T1aN0M0 (number of cases with ipsilateral renal cell carcinoma SPM vs. cases with none-SPM was 82:410) were included for further analysis, 16 RCCs with tumor size more than 4 cm, and 3 cases were T3a RCCs were excluded for a small sample. | | | | | | | | | | | | |
